# Diverse collections in matroids and graphs

**DOI:** 10.1007/s10107-023-01959-z

**Published:** 2023-04-15

**Authors:** Fedor V. Fomin, Petr A. Golovach, Fahad Panolan, Geevarghese Philip, Saket Saurabh

**Affiliations:** 1 University of Bergen, Bergen, Norway; 2 IIT Hyderabad, Kandi, India; 3 Chennai Mathematical Institute, Chennai, India; 4 UMI ReLaX, Chennai, India; 5 Institute of Mathematical Sciences, Chennai, India

**Keywords:** Matroids, Graphs, Diversity of solutions, Parameterized complexity, 68Q27, 05B35, 05C70, 05C85, 68Q25, 68W40

## Abstract

We investigate the parameterized complexity of finding diverse sets of solutions to three fundamental combinatorial problems. The input to the Weighted Diverse Bases problem consists of a matroid $M$, a weight function $\omega :E(M)\rightarrow \mathbb {N}$, and integers $k\ge 1, d\ge 1$. The task is to decide if there is a collection of $k$ *bases* $B_{1}, \dotsc , B_{k}$ of $M$ such that the weight of the symmetric difference of any pair of these bases is at least $d$. The input to the Weighted Diverse Common Independent Sets problem consists of two matroids $M_{1},M_{2}$ defined on the same ground set $E$, a weight function $\omega :E\rightarrow \mathbb {N}$, and integers $k\ge 1, d\ge 1$. The task is to decide if there is a collection of $k$ *common independent sets* $I_{1}, \dotsc , I_{k}$ of $M_{1}$ and $M_{2}$ such that the weight of the symmetric difference of any pair of these sets is at least $d$. The input to the Diverse Perfect Matchings problem consists of a graph $G$ and integers $k\ge 1, d\ge 1$. The task is to decide if $G$ contains $k$ *perfect matchings* $M_{1},\dotsc ,M_{k}$ such that the symmetric difference of any two of these matchings is at least $d$. We show that none of these problems can be solved in polynomial time unless ${{\,\mathrm{\textsf{P}}\,}} ={{\,\mathrm{\textsf{NP}}\,}}$. We derive fixed-parameter tractable (${{\,\mathrm{\textsf{FPT}}\,}}$) algorithms for all three problems with $(k,d)$ as the parameter, and present a $poly(k,d)$-sized kernel for Weighted Diverse Bases.

## Introduction

In this work we study the parameterized complexity of finding *diverse collections of solutions* to three basic algorithmic problems. Two of these problems arise in the theory of matroids. The third problem belongs to the domain of graph theory, and its restriction to bipartite graphs can be rephrased as a question about matroids. Each of these is a fundamental algorithmic problem in its respective domain.

*Diverse* ${{\,\mathrm{\textsf{FPT}}\,}}$  *Algorithms.*  Nearly every existing approach to solving algorithmic problems focuses on finding *one solution of good quality* for a given input. For algorithmic problems which are—eventually—motivated by problems from the real world, finding “one good solution” may not be of much use for practitioners of the real-world discipline from which the problem was originally drawn. This is primarily because the process of abstracting out a “nice” algorithmic problem from a “messy” real-world problem invariably involves throwing out a lot of “side information” which is very relevant to the real-world problem, but is inconvenient, difficult, or even impossible to model mathematically. The other extreme of enumerating *all* (or even all minimal or maximal) solutions to an input instance is also usually not a viable solution. A third approach is to look for *a few solutions of good quality* which are “far away” from one another according to an appropriate notion of distance. The intuition is that given such a collection of “diverse” solutions, an end-user can choose one of the solutions by factoring in the “side information” which is absent from the algorithmic model.

These and other considerations led Fellows to propose *the Diverse X* *Paradigm* [11]. Here “*X*” is a placeholder for an optimization problem, and the goal is to study the fixed-parameter tractability of finding a diverse collection of good-quality solutions for *X*. Recall that the *Hamming distance* of two sets is the size of their symmetric difference. A natural measure of diversity for problems whose solutions are subsets of some kind is the *minimum* Hamming distance of any pair of solutions. In this work we study the parameterized complexity of finding diverse collections of solutions for three fundamental problems with this diversity measure and its weighted variant.

*Our problems.*  Let *M* be a matroid on ground set *E*(*M*) and with rank function *rank*(). The departure point of our work is the classical theorem of Edmonds from 1965 [8] about matroid partition. This theorem states that a matroid *M* has *k* *pairwise disjoint* bases if and only if, for every subset *X* of *E*(*M*),

$$\begin{aligned} k \cdot rank(X) +|E(M) -X|\ge k \cdot rank(M). \end{aligned}$$

An important algorithmic consequence of this result is that given access to an independence oracle for a matroid *M*, one can find a maximum number of *pairwise disjoint bases* of *M* in polynomial time (see, e.g., [23, Theorem 42.5]). This in turn implies, for instance, that the maximum number of pairwise edge-disjoint spanning trees of a connected graph can be found in polynomial time.

We take a fresh look at this fundamental result of Edmonds: what happens if we don’t insist that the bases be pairwise disjoint, and instead allow them to have some pairwise intersection? We work in the weighted setting where each element *e* of the ground set *E*(*M*) has a positive integral weight $\omega (e)$ associated with it, and the weight of a subset *X* of *E*(*M*) is the sum of the weights of the elements in *X*. The relaxed version of the pairwise disjoint bases problem is then: Given an independence oracle for a matroid *M* and integers *k*, *d* as input, find if *M* has *k* bases $B_{1}, \dotsc , B_{k}$ such that for every pair of bases $B_{i}, B_{j}\;;\;i\ne j$ the weight $\omega (B_{i} \bigtriangleup B_{j})$ of their symmetric difference is at least *d*. We call this the Weighted Diverse Bases problem:
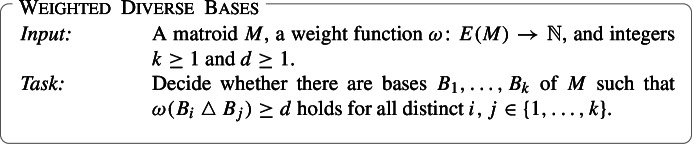
 Due to the expressive power of matroids Weighted Diverse Bases captures many interesting computational problems. We list a few examples; in each case the weight function assigns positive integral weights, $k \ge 1$ and $d \ge 1$ are integers, and we say that a collection of objects is *diverse* if the weight of the symmetric difference of each pair of objects in the collection is at least *d*. When *M* is a graphic matroid Weighted Diverse Bases corresponds to finding diverse *spanning trees* in an edge-weighted graph. When *M* is a vector matroid then this is the problem of finding diverse *column (or row) bases* of a matrix with column (or row) weights. And when *M* is a transversal matroid on a weighted ground set then this problem corresponds to finding diverse *systems of distinct representatives*.

Another celebrated result of Edmonds is the *Matroid Intersection Theorem* [9] which states that if $M_{1}, M_{2}$ are matroids on a common ground set *E* and with rank functions $rank_{1}, rank_{2}$, respectively, then the size of a largest subset of *E* which is independent in both $M_{1}$ and $M_{2}$ (a *common independent set*) is given by

$$\begin{aligned} \min _{T \subseteq E}(rank_{1}(T) + rank_{2}(E-T)). \end{aligned}$$

Edmonds showed that given access to independence oracles for $M_{1}$ and $M_{2}$, a maximum-size common independent set of $M_{1}$ and $M_{2}$ can be found in polynomial time [9]. This is called the Matroid Intersection problem. Frank [15] found a polynomial-time algorithm for the more general Weighted Matroid Intersection problem where the input has an additional weight function $\omega :E\rightarrow \mathbb {N}$ and the goal is to find a common independent set of the maximum *weight*. The second problem that we address in this work is a “diverse” take on Weighted Matroid Intersection where we replace the maximality requirement on individual sets with a lower bound on the weight of their symmetric difference. Given $M_{1}, M_{2},\omega$ as above and integers *k*, *d*, we ask if there are *k* common independent sets whose pairwise symmetric differences have weight at least *d* each; this is the Weighted Diverse Common Independent Sets problem.
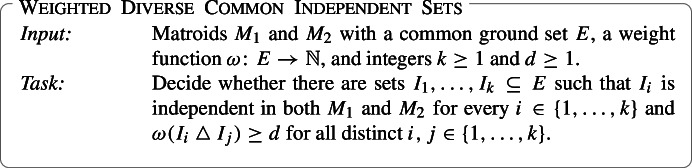
Weighted Diverse Common Independent Sets also captures many interesting algorithmic problems. In particular, the very basic problem of factorizing the common ground set of two matroids introduced by Harvey, Király, and Lau [19] is a special case of our problem. The task of the factorizing problem is, given two matroids $M_1$ and $M_2$ with a common ground set *E*, to decide whether *E* can be partitioned into common bases of $M_1$ and $M_2$. This is equivalent to Weighted Diverse Common Independent Sets with unit weights, where $k=|E|/r$ and $d=2r$ for $r=rank(M_1)=rank(M_2)$. Also the generalization of the factorization problem, where the task is to find a packing of *k* disjoint common bases (see [4] and the refrences therein), is a special case of Weighted Diverse Common Independent Sets. Furthermore, we can give a few examples of more specific problems (see [23, Section 41.1a]). We use “diverse” here in the sense defined in the statement of the problem. Given a bipartite graph *G* with edge weights, Weighted Diverse Common Independent Sets can be used to ask if there is a diverse collection of *k* *matchings* in *G*. A *partial orientation* of an undirected graph *G* is a directed graph obtained by (i) assigning directions to some subset of edges of *G* and (ii) deleting the remaining edges. Given an undirected graph $G = (V,E)$ with edge weights and a function $\iota : V \rightarrow \mathbb {N}$, we say that a partial orientation $\mathcal {O}$ of *G* *respects* $\iota$ if the in-degree of every vertex *v* in $\mathcal {O}$ is at most $\iota (v)$. We can use Weighted Diverse Common Independent Sets to ask if there is a diverse collection of *k* partial orientations of *G*, all of which respect $\iota$. For a third example, let $G = (V, E)$ be an undirected graph with edge weights, in which each edge is assigned a—not necessarily distinct—*color*. A *colorful forest* in *G* is any subgraph of *G* which is a forest in which no two edges have the same color. We can use Weighted Diverse Common Independent Sets to ask if there is a diverse collection of *k* colorful forests in *G*.

Finding whether a bipartite graph has a perfect matching or not is a well-known application of Matroid Intersection ( [23, Section 41.1a]). The third problem that we study in this work is a diverse version of the former problem, extended to general graphs. Note that there is no known interpretation of the problem of finding perfect matchings in (general) undirected graphs in terms of Matroid Intersection.
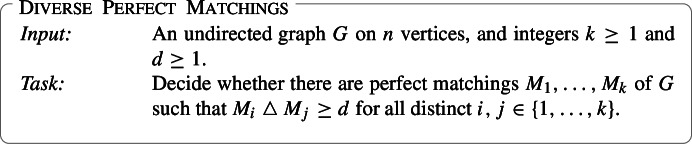


*Our results.*  We assume throughout that matroids in the input are given in terms of an *independence oracle*. Recall that with this assumption, we can find *one* basis of the largest weight and *one* common independent set (of two matroids) of the largest weight, both in polynomial time. In contrast, we show that the diverse versions Weighted Diverse Bases and Weighted Diverse Common Independent Sets are both ${{\,\mathrm{\textsf{NP}}\,}}$-hard, even when the weights are expressed in unary.^1^

### Theorem 1

Both Weighted Diverse Bases and Weighted Diverse Common Independent Sets are strongly ${{\,\mathrm{\textsf{NP}}\,}}$-complete, even for the uniform matroids $U_n^3$.

We observe that the hardness of Weighted Diverse Common Independent Sets also follows from the results of Bérczi and Schwarcz [3] about intractability of finding a packing of common bases in matroids even for unit weights. Furthermore, very recently it was shown by Bérczi, Csáji, and Király [4] that it is already ${{\,\mathrm{\textsf{NP}}\,}}$-hard to find two disjoint common bases of two matroids, when one of the matroids is a partition matroid while the other one is a graphic matroid. Thus, Weighted Diverse Common Independent Sets is already ${{\,\mathrm{\textsf{NP}}\,}}$-hard for unit weights and $k=2$ when restricted the aforementioned matroids.

Given the hardness results, we analyze the parameterized complexity of Weighted Diverse Bases and Weighted Diverse Common Independent Sets with *d*, *k* as the parameters. Our first result is that Weighted Diverse Bases is fixed-parameter tractable (${{\,\mathrm{\textsf{FPT}}\,}}$) under this parameterization:

### Theorem 2

Weighted Diverse Bases can be solved in $2^{\mathcal {O}(dk^2(\log k+\log d))}\cdot |E(M)|^{\mathcal {O}(1)}$ time.

We have a stronger result if the input matroid is given as a representation over a finite field (and not just as a “black box” independence oracle): in this case we show that Weighted Diverse Bases admits a *polynomial kernel* with this parameterization.

### Theorem 3

Given a representation of the matroid *M* over a finite field GF(q) as input, we can compute a kernel of Weighted Diverse Bases of size $\mathcal {O}(k^6d^4\log q)$.

We then show that our second matroid-related diverse problem is also ${{\,\mathrm{\textsf{FPT}}\,}}$ under the same parameterization.

### Theorem 4

Weighted Diverse Common Independent Sets can be solved in $2^{\mathcal {O}(k^3d^2\log (kd))}\cdot |E|^{\mathcal {O}(1)}$ time.

We now turn to the problem of finding diverse perfect matchings. Diverse Perfect Matchings is known to be ${{\,\mathrm{\textsf{NP}}\,}}$-hard already when $k=2$ and *G* is a 3-regular graph [12, 20]. Since all perfect matchings of a graph have the same size the symmetric difference of two distinct perfect matchings is at least 2. Setting $d=1$ (or $d=2$) in Diverse Perfect Matchings is thus equivalent to asking whether *G* has at least *k* distinct perfect matchings. Since a bipartite graph on *n* vertices has at most $\frac{n}{2}!$ perfect matchings and since $\log (\frac{n}{2}!) = \mathcal {O}(n \log n)$ we get—using binary search—that there is a polynomial-time Turing reduction from the problem of *counting* the number of perfect matchings in a bipartite graph to Diverse Perfect Matchings instances with $d=1$. Since the former problem is #P-complete [25] we get

### Theorem 5

Diverse Perfect Matchings with $d=1$ cannot be solved in time polynomial in $n=|V(G)|$ even when graph *G* is bipartite, unless ${{\,\mathrm{\textsf{P}}\,}} = {{\,\mathrm{\textsf{NP}}\,}}$.

Thus we get that Diverse Perfect Matchings is unlikely to have a polynomial-time algorithm even if *one* of the two numbers *k*, *d* is a small constant. We show that the problem *does* have a (randomized) polynomial-time algorithm when *both* these parameters are bounded; Diverse Perfect Matchings is (randomized) ${{\,\mathrm{\textsf{FPT}}\,}}$ with *k* and *d* as parameters:

### Theorem 6

There is an algorithm that given an instance of Diverse Perfect Matchings, runs in time $2^{2^{\mathcal {O}(kd)}}n^{\mathcal {O}(1)}$ and outputs the following: If the input is a No-instance then the algorithm outputs No. Otherwise the algorithm outputs Yes with probability at least $1-\frac{1}{e}$.

Note that Theorem 6 implies, in particular, that Diverse Perfect Matchings can be solved in (randomized) polynomial time when $kd \le c_{1} + \frac{\log \log n}{c_{2}}$ holds for some constants $c_{1}, c_{2}$ which depend on the constant hidden by the $\mathcal {O}()$ notation.

*Our methods.*  We prove the ${{\,\mathrm{\textsf{NP}}\,}}$-hardness results (Theorem 1) by reduction from the 3-Partition problem. To show that Weighted Diverse Bases is ${{\,\mathrm{\textsf{FPT}}\,}}$ (Theorem 2) we observe first that if the input matroid *M* contains a set of size $\Omega (kd)$ which is *both* independent *and* co-independent in *M* then the input is a Yes instance of Weighted Diverse Bases (Lemma 1). We can check for the existence of such a set in time polynomial in |*E*(*M*)|, so we assume without loss of generality that no such set exists. We then show that starting with an arbitrary basis of *M* and repeatedly applying the greedy algorithm (Proposition 2) *poly*(*k*, *d*)-many times we can find, in time polynomial in $(|E(M)| + k + d)$, (i) a subset $S^{*} \subseteq E(M)$ of size *poly*(*k*, *d*) and (ii) a matroid $\widetilde{M}$ on the ground set $S^{*}$ such that $(\widetilde{M},\omega ,k,d)$ is *equivalent* to the input instance $(M,\omega ,k,d)$ (Lemma 2). We also show how to compute a useful partition of $E(\widetilde{M}) = S^{*}$ which speeds up the subsequent ${{\,\mathrm{\textsf{FPT}}\,}}$-time search for a diverse set of bases in $\widetilde{M}$. The kernelization result for Weighted Diverse Bases (Theorem 3) follows directly from Lemma 2. This “compression lemma” is thus the main technical component of our algorithms for Weighted Diverse Bases.

To show that Weighted Diverse Common Independent Sets is ${{\,\mathrm{\textsf{FPT}}\,}}$ (Theorem 4) we observe first that if the two input matroids $M_{1},M_{2}$ have a *common* independent set of size $\Omega (kd)$ then the input is a Yes instance of Weighted Diverse Common Independent Sets (Lemma 3). So we assume that this is not the case, and then show (Lemma 4) that we can construct, in *f*(*k*, *d*) time, a collection $\mathcal {F}$ of common independent sets of $M_1$ and $M_2$ of size *g*(*k*, *d*) such that if the input is a Yes-instance then it has a solution $I_1,\ldots ,I_k$ with $I_i\in \mathcal {F}$ for $i\in \{1,\ldots ,k\}$. The ${{\,\mathrm{\textsf{FPT}}\,}}$ algorithm for Weighted Diverse Common Independent Sets follows by a simple search in the collection $\mathcal {F}$.

Our algorithm for Diverse Perfect Matchings is based on two procedures.

1. Given an undirected graph *G* on *n* vertices, perfect matchings $M_1,\ldots , M_r$ of *G*, and a non-negative integer *s* as input, this procedure (Lemma 6) runs in time $2^{\mathcal {O}(rs)} n^{\mathcal {O}(1)}$ and outputs a perfect matching *M* of *G* such that $\vert M \bigtriangleup M_i\vert \ge 2\,s$ holds for all $i\in \{1,\ldots ,r\}$ (if such a matching exists), with probability at least $\frac{2}{3}e^{-rs}$.
2. Given an undirected graph *G* on *n* vertices, a perfect matching *M* of *G*, and non-negative integers *r*, *d*, *s*, this procedure(Lemma 7) runs in time $2^{\mathcal {O}(r^2s)} n^{\mathcal {O}(1)}$, and outputs *r* perfect matchings $M_1^{\star },\ldots ,M_r^{\star }$ of *G* such that $\vert M \bigtriangleup M_i^{\star }\vert \le s$ holds for all $i\in \{1,\ldots ,r\}$ and $\vert M_i^{\star } \bigtriangleup M_j^{\star } \vert \ge d$ holds for all distinct $i,j\in [r]$ (if such matchings exist), with probability at least $e^{-rs}$. If no such perfect matchings exist, then the algorithm outputs No.

Let (*G*, *k*, *d*) be the input instance of Diverse Perfect Matchings. We use procedure **P1** to greedily compute a collection of matchings which are “far apart”: We start with an arbitrary perfect matching $M_1$. In step *i*, we have a collection of perfect matchings $M_1,\ldots ,M_{i-1}$ such that $\vert M_{j}\bigtriangleup M_{j'}\vert \ge 2^{k-i}d$ holds for any two distinct $j,j'\in \{1,\ldots ,i-1\}$. We now run procedure **P1** with $r=i-1$ and $s=2^{k-i}d$ to find—if it exists—a matching $M_{i}$ such that $\vert M_{i}\bigtriangleup M_j\vert \ge 2^{k-i+1}d$ holds for all $j\in \{1,\ldots ,i\}$. By exhaustively applying **P1** we get a collection of perfect matchings $M_1,\ldots ,M_q$ such that

- for any two distinct integers $i,j\in \{1,\ldots ,q\}$, $|M_i\bigtriangleup M_j|\ge 2^{k-q+1}d$, and
- for any other perfect matching $M\notin \{M_1,\ldots ,M_q\}$ and for any $i\in \{1,\ldots ,q\}$, $\vert M\bigtriangleup M_{i}\vert \le 2^{k-q}d$.

Thus, if $k\le q$, then clearly $\{M_1,\ldots ,M_k\}$ is a solution. Otherwise, let $\mathcal{M}=\{M_1^{\star },\ldots ,M_{k}^{\star }\}$ be a hypothetical solution. Then for each $M_{i}^{\star }$ there is a *unique* matching $M_j$ in $\{M_{1},\ldots ,M_q\}$ such that $\vert M_j\bigtriangleup M_{i}^{\star }\vert < 2^{(k-q)}d$ holds (Proposition 14). For each $i\in \{1,\ldots ,q\}$ we guess the number $r_i$ of perfect matchings from $\mathcal{M}$ that are *close* to $M_i$, and use procedure **P2** to compute a set of $r_i$ diverse perfect matchings that are close to $M_i$. The union of all the matchings computed for all $i\in \{1,\ldots ,q\}$ form a solution.

We use algebraic methods and color coding to design procedure **P1**. The Tutte matrix **A** of an undirected graph *G* over the field ${{\mathbb {F}}}_2[X]$ is defined as follows, where ${{\mathbb {F}}}_2$ is the Galois field on $\{0,1\}$ and $X=\{x_e ~:~ e\in E(G)\}$. The rows and columns of **A** are labeled with *V*(*G*) and for each $e=\{u,v\}\in E(G)$, ${\textbf {A}} [u,v]={\textbf {A}} [v,u]=x_e$. All other entries in **A** are zeros. There is a bijective correspondence between the set of monomials of $det({\textbf {A}})$ and the set of perfect matchings of *G*. Procedure **P1** extracts the required matching from $det({\textbf {A}})$ using color coding. Procedure **P2** is realized using color coding and dynamic programming.

*Related work.*  Recall that all bases of a matroid have the same size, and that the number of bases of a matroid on ground set *E* is at most $2^{|E|}$. So using the same argument as for Theorem 5 we get that Weighted Diverse Bases generalizes—via Turing reductions—the problem of *counting the number of bases* of a matroid. Each of these reduced Weighted Diverse Bases instances will have $d=1$, and a weight function which assigns the weight 1 to each element in the ground set. Counting the number of bases of a matroid is known to be #P-complete even for restricted classes of matroids such as transversal [5], bircircular [17], and binary matroids [26]. Hence we have the following alternative^2^ hardness result for Weighted Diverse Bases

### Theorem 7

Weighted Diverse Bases cannot be solved in time polynomial in |*E*(*M*)| unless ${{\,\mathrm{\textsf{P}}\,}} = {{\,\mathrm{\textsf{NP}}\,}}$, even when $d=1$ and every element of the ground set *E*(*M*) has weight 1.

The study of the parameterized complexity of finding diverse sets of solutions is a recent development, and only a handful of results are currently known. In the work which introduced this notion Baste et al. [1] show that diverse variants of a large class of graph problems which are ${{\,\mathrm{\textsf{FPT}}\,}}$ when parameterized by the *treewidth* of the input graph, are also ${{\,\mathrm{\textsf{FPT}}\,}}$ when parameterized by the treewidth and the number of solutions in the collection. In a second article [2] the authors show that for each fixed positive integer *d*, two diverse variants—one with the *minimum* Hamming distance of any pair of solutions, and the other with the *sum* of all pairwise Hamming distances of solutions—of the *d*-Hitting Set problem are ${{\,\mathrm{\textsf{FPT}}\,}}$ when parameterized by the size of the hitting set and the number of solutions. In a recent article on diverse ${{\,\mathrm{\textsf{FPT}}\,}}$ algorithms [12] the authors show that the problem of finding *two* maximum-sized matchings in an undirected graph such that their symmetric difference is at least *d*, is ${{\,\mathrm{\textsf{FPT}}\,}}$ when parameterized by *d*. Note that our result on Diverse Perfect Matchings generalizes this to $k \ge 2$ matchings, *provided* the input graph has a perfect matching.

In a recent manuscript Hanaka et al. [18] propose a number of results about finding diverse solutions. We briefly summarize their results which are germane to our work. For a collection of sets $X_{1}, \dotsc , X_{k}$ let $d_{sum}(X_{1}, \dotsc , X_{k})$ denote the sum of all pairwise Hamming distances of these sets and let $d_{min}(X_{1}, \dotsc , X_{k})$ denote the smallest Hamming distance of any pair of sets in the collection. Hanaka et al. show that there is an algorithm which takes an independence oracle for a matroid *M* and an integer *k* as input, runs in time polynomial in $(|E(M)| + k)$, and finds a collection $B_{1}, B_{2}, \dotsc , B_{k}$ of *k* bases of *M* which maximizes $d_{sum}(B_{1}, B_{2}, \dotsc , B_{k})$. This result differs from our work on Weighted Diverse Bases in two key aspects. They deal with the unweighted (counting) case, and their diversity measure is the *sum* of the pairwise symmetric differences, whereas we look at the *minimum* (weight of the) symmetric difference. These two measures are, in general, not comparable.

Hanaka et al. also look at the complexity of finding *k* matchings $M_{1}, \dotsc , M_{k}$ in a graph *G* where each $M_{i}$ is of size *t*. They show that such collections of matchings maximizing $d_{min}(M_{1}, \dotsc , M_{k})$ and $d_{sum}(M_{1}, \dotsc , M_{k})$ can be found in time $2^{\mathcal {O}(kt\log (kt))}\cdot |V(G)|^{\mathcal {O}(1)}$. The key difference with our work is that their algorithm looks for matchings of a specified size *t* whereas ours looks for perfect matchings, of size $t = \frac{|V(G)|}{2}$; note that this *t* does not appear in the exponential part of the running time of our algorithm (Theorem 6). The manuscript [18] has a variety of other interesting results on diverse ${{\,\mathrm{\textsf{FPT}}\,}}$ algorithms as well.

As we already mentioned above, the problem of factorizing the ground set into common bases of two matroids or, more generally, of finding a packing of common bases is a special case of Weighted Diverse Common Independent Sets. We refer for the discussion of the results about these problems and their variants to [3, 4, 19, 21].

*Organization of the rest of the paper.*  In the next section we collect together some definitions and preliminary results. In Sect. 3 we prove that Weighted Diverse Bases and Weighted Diverse Common Independent Sets are strongly ${{\,\mathrm{\textsf{NP}}\,}}$-hard. In Sect. 4 we derive our ${{\,\mathrm{\textsf{FPT}}\,}}$ and kernelization algorithms for Weighted Diverse Bases, and in Sect. 5 we show that Weighted Diverse Common Independent Sets is ${{\,\mathrm{\textsf{FPT}}\,}}$. We derive our results for Diverse Perfect Matchings in Sect. 6. We conclude in Sect. 7.

## Preliminaries

For a given universal set *U* and a subset $X\subseteq U$ of *U*, the *complement* of *X* with respect to *U* is the set of all elements of *U* that are *not* in *X*. We use $X \bigtriangleup Y$ to denote the *symmetric difference* $(X \setminus Y)\cup (Y \setminus X)$ of sets *X* and *Y*. We use $\mathbb {N}$ to denote the set of positive integers.

*Parameterized complexity.*   A parameterized problem $\Pi$ is a subset of $\Sigma ^*\times {{\mathbb {N}}}$, where $\Sigma$ is a finite alphabet. We say that a parameterized problem $\Pi$ is *fixed parameter tractable* (${{\,\mathrm{\textsf{FPT}}\,}}$), if there is an algorithm that given an instance (*x*, *k*) of $\Pi$ as input, solves it in time $f(k)\vert x\vert ^{\mathcal {O}(1)}$, where *f* is an arbitrary function and $\vert x\vert$ is the length of *x*. A kernelization algorithm for a parameterized problem $\Pi$ is a polynomial time algorithm (computable function) $\mathcal{A}~:~ \Sigma ^*\times {{\mathbb {N}}}\rightarrow \Sigma ^*\times {{\mathbb {N}}}$ such that $(x,k)\in \Pi$ if and only if $(x',k')=\mathcal{A}((x,k))\in \Pi$ and $\vert x'\vert +k'\le g(k)$ for some computable function *g*. When *g* is a polynomial function, we say that $\Pi$ admits a polynomial kernel. For a detailed overview about parameterized complexity we refer to the monographs [6, 7, 14].

*Matroids.*   We give a brief description of the matroid-related notions that we need. See the book of Oxley [22] for a detailed introduction to matroids. A pair $M=(E,\mathcal {I})$, where *E* is a finite *ground* set and $\mathcal {I}$ is a family of subsets of the ground set, called *independent sets* of *M*, is a *matroid* if it satisfies the following conditions, called *independence axioms*:

1. $$\emptyset \in \mathcal {I}$$.
2. If $A\subseteq B\subseteq E(M)$ and $B\in \mathcal {I}$ then $A\in \mathcal {I}$.
3. If $A, B \in \mathcal {I}$ and $|A| < |B|$, then there is $e \in B \setminus A$ such that $A\cup \{e\} \in \mathcal {I}$.

We use *E*(*M*) and $\mathcal {I}(M)$ to denote the ground set and the set of independent sets, respectively. As is standard for matroid problems, we assume that each matroid *M* that appears in the input is given by an *independence oracle*, that is, an oracle that in constant (or polynomial) time replies whether a given $A\subseteq E(M)$ is independent in *M* or not. An inclusion-wise maximal independent set *B* is called a *basis* of *M*. We use $\mathcal {B}(M)$ to denote the set of bases of *M*. The bases satisfy the following properties, called *basis axioms*:

1. $$\mathcal {B}(M)\ne \emptyset $$.
2. If $B_1,B_2\in \mathcal {B}(M)$, then for every $x\in B_1\setminus B_2$, there is $y\in B_2{\setminus } B_1$ such that $(B_1{\setminus }\{x\})\cup \{y\}\in \mathcal {B}(M)$.

All the bases of *M* have the same size that is called the *rank* of *M*, denoted $\textsf {rank}(M)$. The *rank* of a subset $A\subseteq E(M)$, denoted $\textsf {rank}(A)$, is the maximum size of an independent set $X\subseteq A$; the function $\textsf {rank}:2^{E(M)}\rightarrow \mathbb {Z}$ is the *rank* function of *M*. A set $A\subseteq E(M)$ *spans* an element $x\in E(M)$ if $\textsf {rank}(A\cup \{x\})=\textsf {rank}(A)$. The *closure* (or *span*) of *A* is the set $\textsf {cl}(A)=\{x\in E(M)\mid A\text { spans }x\}$. Closures satisfy the following properties, called *closure axioms*:

1. For every $$A\subseteq E(M)$$, $$A\subseteq \textsf {cl}(A)$$.
2. If $$A\subseteq B\subseteq E(M)$$, then $$\textsf {cl}(A)\subseteq \textsf {cl}(B)$$.
3. For every $$A\subseteq E(M)$$, $$\textsf {cl}(A)=\textsf {cl}(\textsf {cl}(A))$$.
4. For every $A\subseteq E(M)$ and every $x\in E(M)$ and $y\in \textsf {cl}(A\cup \{x\}){\setminus } \textsf {cl}(A)$, $x\in \textsf {cl}(A\cup \{y\})$.

The *dual* of a matroid $M=(E,\mathcal {I})$, denoted $M^{*}$, is the matroid whose ground set is *E* and whose set of bases is $\mathcal {B}^*=\{\overline{B}\mid B\in \mathcal {B}(M)\}$. That is, the bases of $M^*$ are exactly the complements of the bases of *M*, with respect to the ground set *E*. A basis (independent set, rank, respectively) of $M^*$ is a *cobasis* (*coindependent set*, *corank*, respectively) of *M*. We use $\mathcal {I}^*(M)$ to denote the set of coindependent sets of *M*. Also, $\textsf {corank}(M)$ denotes the corank of *M* and $\textsf {corank}(A)$ denotes the corank of a set $A\subseteq E(M)$; $\textsf {corank}(A)$ is the rank of set *A* in the dual matroid $M^{*}$, and $\textsf {corank}(A) = |A| - \textsf {rank}(M) + \textsf {rank}(E \setminus A)$. Given an independence oracle for *M* we can construct—using the augmentation property **(I3)** and with an overhead which is polynomial in |*E*|—a *rank* oracle for *M*, and thence *corank* and *coindependence* oracles for *M*.

For $e\in E(M)$, the matroid $M'=M-e$ is obtained by *deleting* *e* if $E(M')=E(M){\setminus }\{e\}$ and $\mathcal {I}(M')=\{X\in \mathcal {I}(M)\mid e\notin X\}$. It is said that $M'=M/e$ is obtained by *contracting* *e* if $M'=(M^{*}-e)^*$. In particular, if *e* is not a *loop* (i.e., if $\{e\}$ is independent) in *M*, then $\mathcal {I}(M')=\{X{\setminus }\{e\}\mid e\in X\in \mathcal {I}(M)\}$. Notice that deleting an element in *M* corresponds to contracting it in $M^*$ and vice versa; more precisely: $(M-e)^{*} = M^{*}/e$. Let $X\subseteq E(M)$. Then $M-X$ denotes the matroid obtained from *M* by the deletion of the elements of *X* and *M*/*X* is the matroid obtained by consecutive contractions of the elements of *X*. Note that an independence oracle for *M* can itself act as an independence oracle for $M-X$ if we restrict our queries to subsets of $E(M) {\setminus } X$. Let $\textsf {rank}_{M/X}$ denote the rank function of the matroid *M*/*X*. Then for any $Y\subseteq (E(M) {\setminus } X)$ we have that $\textsf {rank}_{M/X}(Y) = \textsf {rank}(X \cup Y) - \textsf {rank}(X)$ [22, 3.1.7]. Given an independence oracle for *M* we can thus easily construct an independence oracle for *M*/*X*.

Let *M* be a matroid and let $\mathbb {F}$ be a field. An $n\times m$-matrix ${\textbf {A}}$ over $\mathbb {F}$ is a *representation of M* *over* $\mathbb {F}$ if there is one-to-one correspondence *f* between *E*(*M*) and the set of columns of ${\textbf {A}}$ such that for any $X\subseteq E(M)$, $X\in \mathcal {I}(M)$ if and only if the columns *f*(*X*) are linearly independent (as vectors of $\mathbb {F}^n$); if *M* has such a representation, then it is said that *M* has a *representation over* $\mathbb {F}$. In other words, ${\textbf {A}}$ is a representation of *M* if *M* is isomorphic to the *linear matroid* of ${\textbf {A}}$, i.e., the matroid whose ground set is the set of columns of ${\textbf {A}}$ and a set of columns is independent if and only if these columns are linearly independent. Observe that, given a representation ${\textbf {A}}$ of *M*, we can verify whether a set is independent by checking the linear independence of the corresponding columns of ${\textbf {A}}$. Hence, we don’t need an explicit independence oracle in this case.

Let $1\le r\le n$ be integers. We use $U_n^r$ to denote the *uniform* matroid of rank *r*, that is, the matroid with the ground set of size *n* such that the bases are all *r*-element subsets of the ground set.

We use the classical results of Edmonds [9] and Frank [15] about the Weighed Matroid Intersection problem. The task of this problem is, given two matroids $M_1$ and $M_2$ with the same ground set *E* and a weight function $\omega :E\rightarrow \mathbb {N}$, find a set *X* of maximum weight such that *X* is independent in both matroids. Edmonds [9] proved that the problem can be solved in polynomial time for the unweighted case (that is, the task is to find a common independent set of maximum size; we refer to this variant as Matroid Intersection) and the result was generalized for the variant with the weights by Frank in [15].

### Proposition 1

([9, 15]) Weighted Matroid Intersection can be solved in polynomial time.

We also need another classical result of Edmonds [10] that a basis of maximum weight can be found by the greedy algorithm. Recall that, given a matroid *M* with a weight function $\omega :E(M)\rightarrow \mathbb {N}$, the greedy algorithm finds a basis *B* of maximum weight as follows. Initially, $B:=\emptyset$. Then at each iteration, the algorithm finds an element of $x\in E(M)\setminus B$ of maximum weight such that $B\cup \{x\}$ is independent and sets $B:=B\cup \{x\}$. The algorithms stops when there is no element that can be added to *B*.

### Proposition 2

([10]) The greedy algorithm finds a basis of maximum weight of a weighted matroid in polynomial time.

We need the following observation (See [22, Lemma 2.1.10]).

### Observation 1

Let *X* and *Y* be disjoint sets such that *X* is independent and *Y* is coindependent in a matroid *M*. Then there is a basis *B* of *M* such that $X\subseteq B$ and $Y\cap B=\emptyset$.

Observe that for any sets *X* and *Y* that are subsets of the same universe, $X\bigtriangleup Y=\overline{X}\bigtriangleup \overline{Y}$. This implies the following.

### Observation 2

For every matroid *M*, every weight function $\omega :E(M)\rightarrow \mathbb {N}$, and all integers $k\ge 1$ and $d\ge 1$, the instances $(M,\omega ,k,d)$ and $(M^*,\omega ,k,d)$ of Weighted Diverse Bases are equivalent.

## Hardness of Weighted Diverse Bases and Weighted Diverse Common Independent Sets

We show that Weighted Diverse Bases and Weighted Diverse Common Independent Sets are ${{\,\mathrm{\textsf{NP}}\,}}$-complete in the strong sense even for uniform matroids.

**Theorem 1** *Both* Weighted Diverse Bases *and* Weighted Diverse Common Independent Sets *are strongly* ${{\,\mathrm{\textsf{NP}}\,}}$ *-complete, even for the uniform matroids* $U_n^3$.

### Proof

We prove the claim for Weighted Diverse Bases by a reduction from the 3-Partition problem. The input to 3-Partition consists of a positive integer *b* and a multiset $S=\{s_1,\ldots ,s_{3n}\}$ of 3*n* positive integers such that (i) $\frac{b}{4}< s_{i}< \frac{b}{2}$ holds for each $i\in \{1,\ldots ,3n\}$ and (ii) $\sum _{i=1}^{3n}s_{i} = nb$. The task is to decide whether *S* can be partitioned into *n* multisets $S_1,\ldots ,S_n$ such that $\sum _{s \in S_{i}}s = b$ holds for each $S_{i}$. Note that each multiset $S_{i}$ in such a partition must contain exactly three elements from *S*. This problem is known to be ${{\,\mathrm{\textsf{NP}}\,}}$-complete in the strong sense, i.e., it is ${{\,\mathrm{\textsf{NP}}\,}}$-complete even if the input integers are encoded in unary [16, SP15].

Let $(b, S=\{s_1,\ldots ,s_{3n})\}$ be an instance of 3-Partition with $n\ge 3$. We set *M* to be the uniform matroid $U_{3n}^3$ on the ground set $\{1,\ldots ,3n\}$, and define the weight function to be $\omega (i)=s_i$ for $i\in \{1,\ldots ,3n\}$. We set $d=2b$. We will now show that (*b*, *S*) is a yes-instance of 3-Partition if and only if $(M,\omega ,n,d)$ is a yes-instance of Weighted Diverse Bases.

In the forward direction, suppose that $S_1,\ldots ,S_n$ is a partition of *S* into triples of integers such that the sum of elements of each $S_i$ is *b*. Let $B_1,\ldots ,B_n$ be the corresponding partition of $\{1,\ldots ,3n\}$, that is, $B_i=\{i_1,i_2,i_3\}$ if and only if $S_i=\{s_{i_1},s_{i_2},s_{i_3}\}$ for each $i\in \{1,\ldots ,n\}$. Clearly, $B_1,\ldots ,B_n$ are pairwise disjoint bases of *M*. Then for every distinct $i,j\in \{1,\ldots ,n\}$, $\omega (B_i\bigtriangleup B_j) = \omega (B_{i}) + \omega (B_{j}) = 2b$. Therefore, $(M,\omega ,n,d)$ is a yes-instance of Weighted Diverse Bases.

In the reverse direction, assume that $(M,\omega ,n,d)$ is a yes-instance of Weighted Diverse Bases. Let $B_1,\ldots ,B_n$ be bases of *M* such that $\omega (B_i\bigtriangleup B_j)\ge d=2b$ for distinct $i,j\in \{1,\ldots ,n\}$.

We claim that $B_1,\ldots ,B_n$ are pairwise disjoint. For the sake of contradiction, assume that there are distinct $i,j\in \{1,\ldots ,n\}$ such that $B_i\cap B_j\ne \emptyset$. Let $X_1=B_i{\setminus } B_j$ and $X_2=B_j{\setminus } B_i$. Note that $|X_1|\le 2$ and $|X_2|\le 2$. We have that $\omega (X_1)=\sum _{h\in X_1}\omega (h)=\sum _{h\in X_1}s_h<|X_1|b/2 < b$. Similarly, $\omega (X_2)<b$. Therefore, $\omega (B_i\bigtriangleup B_j)=\omega (X_1)+\omega (X_2)<2b$; a contradiction. We conclude that the bases $B_1,\ldots ,B_n$ are pairwise disjoint. This implies that $B_1,\ldots ,B_n$ is a *partition* of $\{1, 2, \dotsc , 3n\}$.

Next we show that $\omega (B_i)=b$ holds for every $i\in \{1,\ldots ,n\}$. Suppose that there is an $h\in \{1,\ldots ,n\}$ such that $\omega (B_h)>b$. Let $I=\{1,\ldots ,3n\}{\setminus } B_h$ and $J=\{1,\ldots ,n\}{\setminus }\{h\}$. We have that $\sum _{i\in I}\omega (i)<\sum _{i=1}^{3n}\omega (i)-b=b(n-1)$. Since $B_1,\ldots ,B_{h-1},B_{h+1}\ldots ,B_n$ form a partition of *I*, we get that $\sum _{i\in J}\omega (B_i)<b(n-1)$ holds as well. Recall that $n\ge 3$. Then

$$\begin{aligned} \sum _{ \{i,j\}\text { s.t. }i,j\in J,~i\ne j}(\omega (B_i)+\omega (B_j)) \le (n-2)\sum _{i\in J}\omega (B_i)<b(n-1)(n-2). \end{aligned}$$

The first inequality above comes from the fact that since $|J| = n-1$, for each index $i \in J$ the term $\omega (B_{i})$ appears in *at most* $n-2$ terms of the form $(\omega (B_i)+\omega (B_j))$ in the summation on the left hand side. Now suppose $(\omega (B_i)+\omega (B_j)) \ge 2b$ holds for *all* pairs $i,j \in J,\;i\ne j$. Then the sum on the left hand side would be at least $$\left( {\begin{array}{c}|J|\\ 2\end{array}}\right) \cdot 2b = (n-1)(n-2)b$$, a contradiction. Therefore, there must exist distinct $i,j\in J$ such that $\omega (B_i)+\omega (B_j)<2b$ holds. And this contradicts our assumption that $\omega (B_i\bigtriangleup B_j)\ge 2b$ holds for all such *i*, *j*. We conclude that $\omega (B_i)\le b$ holds for every $i\in \{1,\ldots ,n\}$. And since $\sum _{i=1}^n\omega (B_i)=bn$, we get that $\omega (B_i)=b$ holds for every $i\in \{1,\ldots ,n\}$.

Finally, we consider the partition $S_1,\ldots ,S_n$ of *S* corresponding to $B_1,\ldots ,B_i$, that is, for each $B_i=\{i_1,i_2,i_3\}$, we define $S_i=\{s_{i_1},s_{i_2},s_{i_3}\}$. Clearly, $s_{i_1}+s_{i_2}+s_{i_3}=\omega (B_i)=b$. Thus we get that (*b*, *S*) is a yes-instance of 3-Partition. This concludes the proof for Weighted Diverse Bases.

The reduction for Weighted Diverse Common Independent Sets is also from 3-Partition, and is nearly identical to the above reduction for Weighted Diverse Bases. Given an instance $(b, S=\{s_1,\ldots ,s_{3n})\}$ of 3-Partition with $n\ge 3$, we set each of $M_{1}, M_{2}$ to be the uniform matroid $U_{3n}^3$ on the ground set $\{1,\ldots ,3n\}$, and define the weight function to be $\omega (i)=s_i$ for $i\in \{1,\ldots ,3n\}$. We set $d=2b$. We will now show that (*b*, *S*) is a yes-instance of 3-Partition if and only if $(M_{1},M_{2},\omega ,n,d)$ is a yes-instance of Weighted Diverse Common Independent Sets.

In the forward direction, suppose that $S_1,\ldots ,S_n$ is a partition of *S* into triples of integers such that the sum of elements of each $S_i$ is *b*. Let $I_1,\ldots ,I_n$ be the corresponding partition of $\{1,\ldots ,3n\}$, that is, $I_i=\{i_1,i_2,i_3\}$ if and only if $S_i=\{s_{i_1},s_{i_2},s_{i_3}\}$ for each $i\in \{1,\ldots ,n\}$. Clearly, $I_1,\ldots ,I_n$ are pairwise disjoint common independent sets of $M_{1}$ and $M_{2}$, and for every distinct $i,j\in \{1,\ldots ,n\}$, $\omega (I_i\bigtriangleup I_j) = \omega (I_{i}) + \omega (I_{j}) = 2b$. Therefore, $(M_{1},M_{2},\omega ,n,d)$ is a yes-instance of Weighted Diverse Common Independent Sets.

In the reverse direction, assume that $(M_{1},M_{2},\omega ,n,d)$ is a yes-instance of Weighted Diverse Common Independent Sets, and let $I_1,\ldots ,I_n$ be common independent sets of $M_{1}$ and $M_{2}$ such that $\omega (I_i\bigtriangleup I_j)\ge d=2b$ for distinct $i,j\in \{1,\ldots ,n\}$. Since every independent set in the matroids $M_{1},M_{2}$ has at most three elements, and since $s_{i}< \frac{b}{2}$ holds for each $i\in \{1,\ldots ,3n\}$, we get that the sets $I_1,\ldots ,I_n$ are pairwise disjoint. If two of these sets, say $I_{i},I_{j}$ have at most two elements each then $\omega (I_i\bigtriangleup I_j) < 4\cdot \frac{b}{2} = 2b$, a contradiction. So at most one of these sets has at most two elements; every other set in the collection has exactly three elements.

If all the sets $I_1,\ldots ,I_n$ have three elements each then they are a pairwise disjoint collection of *n* *bases* of $M_{1}$, and the argument that we used for the reverse direction in the proof for Weighted Diverse Bases tells us that (*b*, *S*) is a yes-instance of 3-Partition. In the remaining case there is exactly one set of size two among $I_1,\ldots ,I_n$; without loss of generality, let this smaller set be $I_{1}$. Then $|\bigcup _{i=1}^{n}I_{i}| = 3n-1$. Let $x = \{1, 2, \dotsc , 3n\} {\setminus } \bigcup _{i=1}^{n}I_{i}$ be the unique element which is not in any of these independent sets. Then $(I_{1} \cup \{x\}),\ldots ,I_n$ is a pairwise disjoint collection of *n* bases of $M_{1}$ such that the weight of the symmetric difference of any pair of these bases is at least $d=2b$, and the argument that we used for the reverse direction in the proof for Weighted Diverse Bases tells us that (*b*, *S*) is a yes-instance of 3-Partition. $\square$

## An FPT algorithm and kernelization for Weighted Diverse Bases

In this section, we show that Weighted Diverse Bases is ${{\,\mathrm{\textsf{FPT}}\,}}$ when parameterized by *k* and *d*. Moreover, if the input matroid is representable over a finite field and is given by such a representation, then Weighted Diverse Bases admits a polynomial kernel.

We start with the observation that if the input matroid has a sufficiently big set that is simultaneously independent and coindependent, then diverse bases always exist.

### Lemma 1

Let *M* be a matroid, and let $k\ge 1$ and $d\ge 1$ be integers. If there is $X\subseteq E(M)$ of size at least $k\lceil \frac{d}{2}\rceil$ such that *X* is simultaneously independent and coindependent, then $(M,\omega ,k,d)$ is a yes-instance of Weighted Diverse Bases for any weight function $\omega$.

### Proof

Let $X\subseteq E(M)$ be a set of size at least $k\lceil \frac{d}{2}\rceil$ such that *X* is simultaneously independent and coindependent. Then there is a partition $X_1,\ldots ,X_k$ of *X* such that $|X_i|\ge \lceil \frac{d}{2}\rceil$ for every $i\in \{1,\ldots ,k\}$. Let $i\in \{1,\ldots ,k\}$. Since *X* is independent, $X_i$ is independent, and since *X* is coindependent, then $X\setminus X_i$ is coindependent. Then by Observation 1, there is a basis $B_i$ of *M* such that $X_i\subseteq B_i$ and $B_i\cap (X{\setminus } X_i)=\emptyset$. The latter property means that $B_i\cap X_j=\emptyset$ for every $j\in \{1,\ldots ,k\}$ such that $j\ne i$. We consider the bases $B_i$ defined in this manner for all $i\in \{1,\ldots ,k\}$. Then for every distinct $i,j\in \{1,\ldots ,k\}$, $X_i\cup X_j\subseteq B_i\bigtriangleup B_j$. Therefore, $\omega (B_i\bigtriangleup B_j)\ge \omega (X_i\cup X_j)\ge |X_i\cup X_j|=|X_i|+|X_j|\ge 2\lceil \frac{d}{2}\rceil \ge d$ for any $\omega :E(M)\rightarrow \mathbb {N}$. Hence, $(M,\omega ,k,d)$ is a yes-instance of Weighted Diverse Bases.$\square$

Our results are based on the following lemma.

### Lemma 2

There is an algorithm that, given an instance $(M,\omega ,k,d)$ of Weighted Diverse Bases, runs in time polynomial in $(|E(M)| + k + d)$ and either correctly decides that $(M,\omega ,k,d)$ is a yes-instance or outputs an equivalent instance $(\widetilde{M},\omega ,k,d)$ of Weighted Diverse Bases such that $E(\widetilde{M})\subseteq E(M)$ and $|E(\widetilde{M})|\le 2\lceil \frac{d}{2}\rceil ^2k^3$. In the latter case, the algorithm also computes a partition $(L,L^*)$ of $E(\widetilde{M})$ with the property that for every basis *B* of $\widetilde{M}$, $|B\cap L|\le \lceil \frac{d}{2}\rceil k$ and $|L^*{\setminus } B|\le \lceil \frac{d}{2}\rceil k$, and the algorithm outputs an independence oracle for $\widetilde{M}$ that answers queries for $\widetilde{M}$ in time polynomial in |*E*(*M*)|. Moreover, if *M* is representable over a finite field $\mathbb {F}$ and is given by such a representation, then the algorithm outputs a representation of $\widetilde{M}$ over $\mathbb {F}$.

### Proof

Let $(M,\omega ,k,d)$ be an instance of Weighted Diverse Bases. Recall that *M* is given as an independence oracle. We construct an independence oracle for the dual matroid $M^*$, and then solve Matroid Intersection for *M* and $M^*$ using Proposition 1. Let *X* be the set computed by the Matroid Intersection algorithm. Then $X\subseteq E(M)$ is a set of maximum size that is both independent and coindependent in *M*. If $|X|\ge k\lceil \frac{d}{2}\rceil$, then $(M,\omega ,k,d)$ is a yes-instance of Weighted Diverse Bases by Lemma 1; the problem is solved and we return the answer.

Assume from now on that this is not the case, and that $|X|\le k\lceil \frac{d}{2}\rceil -1$ holds. Let *B* be an arbitrary basis of *M*, and let $\overline{B} = (E(M) \setminus B)$. If $\textsf {rank}(\overline{B})\ge k\lceil \frac{d}{2}\rceil$ then there exists an independent set $Y\subseteq \overline{B}$ of size at least $k\lceil \frac{d}{2}\rceil$. But *Y* is also a *coindependent* set of size at least $k\lceil \frac{d}{2}\rceil$, which contradicts our assumption. Thus we get that $\textsf {rank}(\overline{B})\le k\lceil \frac{d}{2}\rceil -1$ and $\textsf {corank}(B)\le k\lceil \frac{d}{2}\rceil -1$ hold for any basis *B* of *M*.

Let $\ell =\lceil \frac{d}{2}\rceil k^2$. Fix an arbitrary basis *B* of *M*. We construct sets $S_0,\ldots ,S_\ell$ iteratively. We set $S_0=B$. For $i\ge 1$ we construct $S_i$ from $S_{i-1}$ as follows. If $E(M){\setminus } S_{i-1}=\emptyset$, we set $X_i=\emptyset$. Otherwise we set $X_{i}$ to be a basis of maximum weight in the matroid $M-S_{i-1}$; we find $X_{i}$ using the greedy algorithm (see Proposition 2). Finally, we set $S_i=S_{i-1}\cup X_i$.

Let $S=S_\ell$ and $L=S{\setminus } B$. Since $\textsf {rank}(\overline{B})\le k\lceil \frac{d}{2}\rceil -1$, we get that *every* independent set contained in the set $\overline{B} = (E(M) \setminus B)$ has size at most $k\lceil \frac{d}{2}\rceil -1$. And since *L* is a disjoint union of $\ell$ such independent sets we get that $|L|=|S{\setminus } B|\le \ell (k\lceil \frac{d}{2}\rceil -1)\le \lceil \frac{d}{2}\rceil ^2k^3$. We now prove the following crucial proposition.

### Proposition 3

If $(M,\omega ,k,d)$ is a yes-instance of Weighted Diverse Bases, then there is a solution, that is, a family of bases $B_1,\ldots ,B_k$ such that $\omega (B_i \bigtriangleup B_j)\ge d$ for all distinct $i,j\in \{1,\ldots ,k\}$, with the property that $B_i\subseteq S$ for every $i\in \{1,\ldots ,k\}$.

### Proof

Let $(M,\omega ,k,d)$ be a yes-instance, and let the family of bases $B_1,\ldots ,B_k$ be a solution which maximizes the size of the set $((\bigcup _{i=1}^{k}B_{i}) \cap S)$ of vertices in the bases which are also in the set *S*. We show that $B_i\subseteq S$ holds for every $i\in \{1,\ldots ,k\}$. The proof is by contradiction. Assume that there is an $h\in \{1,\ldots ,k\}$ such that $B_h{\setminus } S\ne \emptyset$. Recall that $\textsf {rank}(M-B)=\textsf {rank}(\overline{B})\le k\lceil \frac{d}{2}\rceil -1$. Therefore, $|B_i\setminus B|\le k\lceil \frac{d}{2}\rceil -1$ holds for every $i\in \{1,\ldots ,k\}$, and $|\cup _{i=1}^k(B_i{\setminus } B)|\le k(k\lceil \frac{d}{2}\rceil -1)<\ell$. Let $X_1,\ldots ,X_\ell$ be the independent sets used to construct the sets $S_1,\ldots ,S_\ell$. Since $(X_{j} \cap B) =\emptyset$ holds for all $j\in \{1,\ldots , \ell \}$ we get that $(X_{j} \cap B_{i}) = (X_{j} \cap (B_{i} \setminus B))$ holds for all $i\in \{1,\ldots , k\}, j\in \{1,\ldots , \ell \}$. So the number $|\cup _{i=1}^k(B_i\setminus B)|$ of elements from the bases $B_1,\ldots ,B_k$ which could *potentially* be part of any of the sets $X_1,\ldots ,X_\ell$ is strictly less than the number of these latter sets. Hence from the pigeonhole principle we get that there is a $t\in \{1,\ldots ,\ell \}$ such that $X_t\cap B_i=\emptyset$ holds for all $i\in \{1,\ldots ,k\}$. Let $A=B_h\cap S_{t-1}$ and $Y=B_h{\setminus } S_{t-1}$. We show that there is $Z\subseteq X_t$ such that

(i) $B_h'=A\cup Z$ is a basis, and
(ii) $$\omega (Z)\ge \omega (Y)$$.

We construct *Z* by greedily augmenting *A* with elements of $X_t$. Let $\sigma$ be the order in which the greedy algorithm picks elements from the set $E(M) \setminus S_{t-1}$ to add them to the set $X_{t}$. Initially we set $Z:=\emptyset$. Then we select the first $x\in X_t\setminus Z$ in $\sigma$ such that $A\cup Z\cup \{x\}$ is independent, and we set $Z:=Z\cup \{x\}$. We stop when there is no $x\in X_t\setminus Z$ such that $A\cup Z\cup \{x\}$ is independent. We prove that (i) and (ii) are fulfilled for *Z*.

First we show that (i) holds. From the construction we get that $X_{t}$ is a basis of the matroid $M-S_{t-1}$. This implies that $(E(M) {\setminus } S_{t-1}) \subseteq \textsf {cl}(X_{t})$ holds. Now since $Y = (B_{h} {\setminus } S_{t-1})$ is a subset of $(E(M) {\setminus } S_{t-1})$ we get that $Y\subseteq \textsf {cl}(X_t)$ holds. Since $(A \cup Z)$ is independent, and there is no $x\in X_t{\setminus } Z$ such that $A\cup Z\cup \{x\}$ is independent, we get that $X_t\subseteq \textsf {cl}(A\cup Z)$ holds. Now by **(CL2)** and **(CL3)**, $Y\subseteq \textsf {cl}(\textsf {cl}(A\cup Z))=\textsf {cl}(A\cup Z)$. And by **(CL1)**, $A\subseteq \textsf {cl}(A\cup Z)$ and we conclude that $A\cup Y\subseteq \textsf {cl}(A\cup Z)$ holds. But $(A \cup Y) = B_{h}$ is a *basis* of *M*, and so [22, Proposition 1.4.9] $\textsf {cl}(A \cup Y) = E(M)$. Applying **(CL2)** and **(CL3)** we get that $E(M) \subseteq \textsf {cl}(A\cup Z)$ which implies that $\textsf {cl}(A\cup Z) = E(M)$. Now since $A\cup Z$ is independent we get (see, e.g., [22, Section 1.4, Exercise 2]) that $B_h'=A\cup Z$ is a basis.

Now we show that (ii) holds. Let $Z=\{z_1,\ldots ,z_s\}$, where the elements are indexed according to the order in which they are added to *Z* by the greedy augmentation described above. Note that $\omega (z_1)\ge \cdots \ge \omega (z_s)$. Since $B_h$ and $B_h'$ are bases, $Y=(B_{h} {\setminus } S_{t-1})$, and $Z=(B'_{h} {\setminus } S_{t-1})$, we get that $|Y|=|Z|$. Observe also that $Y \cap Z = \emptyset$. We define (i) $Z_0=\emptyset$ and (ii) $Z_i=\{z_1,\ldots ,z_i\}$ for $i\in \{1,\ldots ,s\}$. We show that there is an ordering $\langle y_1,\ldots ,y_s \rangle$ of the elements of *Y* such that the set $A\cup Z_{i-1}\cup \{y_i\}$ is independent for every $i\in \{1,\ldots ,s\}$. We define this order inductively, starting with $y_{s}$ and proceeding in decreasing order of the subscript.

We set $y_{s}$ to be an element $y\in (A\cup Y){\setminus } (A\cup Z_{s-1}) = (Y {\setminus } Z_{s-1})$ such that $A\cup Z_{s-1}\cup \{y\}$ is independent. Since $|A\cup Z_{s-1}|<|A\cup Y|$ we know from **(I3)** such an element must exist. For the inductive step, assume that for some fixed $i\in \{1,\ldots ,s-1\}$ distinct elements $y_{i+1},\ldots ,y_s\in Y$ have been defined such that $A\cup Z_i\cup \{y_{i+1},\ldots ,y_s\}$ is independent. Note that $|A\cup Z_i\cup \{y_{i+1},\ldots ,y_s\}| = |A\cup Y|$. Then $R=A\cup Z_{i-1}\cup \{y_{i+1},\ldots ,y_s\}$ is independent by **(I2)**, and by **(I3)**, there must exist an element $y\in (A\cup Y)\setminus R$ such that $R\cup \{y\}= A\cup Z_{i-1}\cup \{y, y_{i+1},\ldots ,y_s\}$ is independent. We set $y_{i}$ to be this element *y*. Observe that due to **(I2)**, $A\cup Z_{i-1}\cup \{y_i\}$ is indeed independent for every $i\in \{1,\ldots ,s\}$.

We claim that $\omega (y_i)\le \omega (z_i)$ holds for every $i\in \{1,\ldots ,s\}$. For the sake of contradiction, assume that this is not the case and let $i\in \{1,\ldots ,s\}$ be the first index such that $\omega (y_i)>\omega (z_i)$ holds. Recall that $X_t$ is constructed by the greedy algorithm. Denote by $W\subset X_t$ the set of elements that are prior $z_i$ in the ordering $\sigma$. Suppose that $y_i\in \textsf {cl}(W)$. By the construction of *Z*, $W\subseteq \textsf {cl}(A\cup Z_{i-1})$, because $z_i$ is the first element in $\sigma$ such that $A\cup Z_{i-1}\cup \{z_i\}$ is independent. By **(CL2)** and **(CL3)**, we have that $y_i\in \textsf {cl}(\textsf {cl}(A\cup Z_{i-1}))=\textsf {cl}(A\cup Z_{i-1})$. However, this contradicts the property that $A\cup Z_{i-1}\cup \{y_i\}$ is independent. Hence, $y_i\notin \textsf {cl}(W)$. This implies that $W\cup \{y_i\}$ is independent. But this means that the greedy algorithm would have $y_i$ over $z_i$ in the construction of $X_t$, because $\omega (y_i)>\omega (z_i)$; a contradiction. This proves that $\omega (y_i)\le \omega (z_i)$ holds for every $i\in \{1,\ldots ,s\}$. Therefore, $\omega (Z)\ge \omega (Y)$ and (ii) is fulfilled. This completes the proof of the existence of a set $Z\subseteq X_t$ satisfying (i) and (ii).

We replace the basis $B_h$ in the solution by $B_h'=A\cup Z=(B_h{\setminus } Y)\cup Z$. We show that the resulting family of bases is a solution to the instance $(M,\omega ,k,d)$. Clearly, it is sufficient to show that for every $i\in \{1,\dots ,k\}$ such that $i\ne h$, $\omega (B_h'\bigtriangleup B_i)\ge d$, as the other pairs of bases are the same as before. By the choice of $t\in \{1,\ldots ,\ell \}$ we have that $X_{t}\cap B_i=\emptyset$ holds for all $i\in \{1,\dots ,k\}$. And since $Z\subseteq X_t$ we have that $Z\subseteq B_h'\bigtriangleup B_i$ holds. Then, $\omega (B_h'\bigtriangleup B_i)=\omega (((B_h{\setminus } Y)\cup Z)\bigtriangleup B_i))\ge \omega (B_h\bigtriangleup B_i)-\omega (Y)+\omega (Z)\ge \omega (B_h\bigtriangleup B_i)\ge d$ as required. We have that the replacement of $B_h$ by $B_h'$ gives a solution. However $B_h'\subseteq S_t\subseteq S$ whereas $B_{h}{\setminus } S \ne \emptyset$, and this contradicts the assumption that $B_1,\ldots ,B_k$ is a solution such that the number of vertices of the bases in *S* is the maximum. This concludes the proof of the proposition. $\square$

Let $\widehat{M}=M-(E(M){\setminus } S)$. Then $E(\widehat{M}) = S$ and the set *B* is a basis of $\widehat{M}$ as well. Proposition 3 immediately implies the following property.

### Proposition 4

The instances $(M,\omega ,k,d)$ and $(\widehat{M},\omega ,k,d)$ of Weighted Diverse Bases are equivalent.

We now repeat the argument that preceded Proposition 3, this time with the dual matroid $\widehat{M}^{*}$ and starting with its basis $\widehat{B} = (E(\widehat{M}){\setminus } B) = L$. Recall that $\ell =\lceil \frac{d}{2}\rceil k^2$. We construct sets $S^{*}_{0},\ldots ,S^{*}_{\ell }$ iteratively. We set $S^{*}_{0}=\widehat{B}$. For $i\ge 1$ we construct $S^{*}_{i}$ from $S^{*}_{i-1}$ as follows. If $E(\widehat{M}){\setminus } S^{*}_{i-1}=\emptyset$, we set $X^{*}_i=\emptyset$. Otherwise we set $X^{*}_{i}$ to be a basis of maximum weight in the matroid $\widehat{M}^{*}-S^{*}_{i-1}$, which we find using the greedy algorithm. Finally, we set $S^{*}_i=S^{*}_{i-1}\cup X^{*}_i$.

Let $S^{*}=S^{*}_\ell$ and $L^{*} = S^{*}{\setminus } \widehat{B} = S^{*}\cap B$. Since $\textsf {corank}(B)\le k\lceil \frac{d}{2}\rceil -1$, we get that *every* coindependent set contained in the set *B* has size at most $k\lceil \frac{d}{2}\rceil -1$. And since $L^{*}$ is a disjoint union of $\ell$ such coindependent sets we get that $|L^{*}|=|S^{*}\cap B|\le \ell (k\lceil \frac{d}{2}\rceil -1)\le \lceil \frac{d}{2}\rceil ^2k^3$. Restating Proposition 3 for $\widehat{M}^*$, we get that if $(\widehat{M}^*,\omega ,k,d)$ is a yes-instance of Weighted Diverse Bases, then there is a solution, that is, a family of bases $B_1^*,\ldots ,B_k^*$ of $\widehat{M}^*$ such that $\omega (B_i^* \bigtriangleup B_j^*)\ge d$ holds for all distinct $i,j\in \{1,\ldots ,k\}$, with the property that $B_i^*\subseteq S^*$ for every $i\in \{1,\ldots ,k\}$. In terms of $\widehat{M}$, the same property can be stated as follows.

### Proposition 5

If $(\widehat{M},\omega ,k,d)$ is a yes-instance of Weighted Diverse Bases, then there is a solution, that is, a family of bases $B_1,\ldots ,B_k$ such that $\omega (B_i \bigtriangleup B_j)\ge d$ for all distinct $i,j\in \{1,\ldots ,k\}$ with the property that $\overline{B_i}\subseteq S^*$ for every $i\in \{1,\ldots ,k\}$, where $\overline{B_{i}} = (E(\widehat{M}) \setminus B_{i})$.

Since $E(\widehat{M}) = (B \cup \widehat{B})$ and $\widehat{B} \subseteq S^{*} \subseteq E(\widehat{M})$ we have that $E(\widehat{M}) = (B \cup S^{*})$. Hence from Proposition 5 we get that if $(\widehat{M},\omega ,k,d)$ is a yes-instance, then it has a solution $B_1,\ldots ,B_k$ such that $(B{\setminus } S^*)\subseteq B_i$ holds for every $i\in \{1,\ldots ,k\}$. That is, elements from the set $(B\setminus S^*)$ do not contribute to the weight $\omega (B_i \bigtriangleup B_j)$ for any distinct $i,j\in \{1,\ldots ,k\}$. So a transformation that removes the subset $(B\setminus S^*)$ from the ground set of $\widehat{M}$ is safe, *provided that* (i) $B_{i} {\setminus }(B{\setminus } S^{*}) = (B_{i} \cap S^{*})$ is a basis of the resulting matroid for all $i\in \{1,\ldots ,k\}$, and (ii) for *any* basis $B'$ of the resulting matroid, $B' \cup (B{\setminus } S^*)$ is a basis of $\widehat{M}$.

We now show that the operation of *contracting* the set $(B{\setminus } S^{*})$ has both these properties. Let $\widetilde{M}=\widehat{M}/(B{\setminus } S^*)$. Then $E(\widetilde{M}) = (B \cup S^{*}) {\setminus } (B{\setminus } S^*) = S^{*}$. Let $\textsf {rank}(\widehat{M}), \textsf {rank}(\widetilde{M})$ be the ranks and $\widehat{\textsf {rank}}, \widetilde{\textsf {rank}}$ be the rank functions of the two matroids $\widehat{M}, \widetilde{M}$, respectively. Recall that $\widetilde{\textsf {rank}}(X) = \widehat{\textsf {rank}}((B{\setminus } S^*) \cup X) - \widehat{\textsf {rank}}(B{\setminus } S^*)$ holds for all $X \subseteq E(\widetilde{M}) = S^{*}$. Now $\textsf {rank}(\widetilde{M}) = \widetilde{\textsf {rank}}(S^{*}) = \widehat{\textsf {rank}}(B \cup S^{*}) - \widehat{\textsf {rank}}(B {\setminus } S^{*}) = \textsf {rank}(\widehat{M}) - |B {\setminus } S^{*}|$, where the last equation holds because *B* is a basis of $\widehat{M}$. And for any $i\in \{1,\ldots ,k\}$, $\widetilde{\textsf {rank}}(B_{i} \cap S^{*}) = \widehat{\textsf {rank}}((B {\setminus } S^{*}) \cup (B_{i} \cap S^{*})) - \widehat{\textsf {rank}}(B {\setminus } S^{*}) = \textsf {rank}(\widehat{M}) - |B {\setminus } S^{*}| = \textsf {rank}(\widetilde{M})$, where the second equation holds because $B, B_{i}$ are bases of $\widehat{M}$ and $B_{i} \subseteq ((B {\setminus } S^{*}) \cup (B_{i} \cap S^{*}))$. Thus $(B_{i} \cap S^{*})$ is a basis of $\widetilde{M}$. Finally, let $B'$ be an arbitrary basis of $\widetilde{M}$. Then $\widetilde{\textsf {rank}}(B') = \textsf {rank}(\widetilde{M}) = \textsf {rank}(\widehat{M}) - |B {\setminus } S^{*}|$. Rearranging the expression for $\widetilde{\textsf {rank}}(B')$ in terms of $\widehat{\textsf {rank}}$ we get: $\widehat{\textsf {rank}}((B {\setminus } S^{*}) \cup B') = \widetilde{\textsf {rank}}(B') + \widehat{\textsf {rank}}(B {\setminus } S^{*}) = \textsf {rank}(\widehat{M}) - |B {\setminus } S^{*}| + |B {\setminus } S^{*}| = \textsf {rank}(\widehat{M})$ where the second equation holds because *B* is a basis of $\widehat{M}$. Thus $(B {\setminus } S^{*}) \cup B'$ is a basis of $\widehat{M}$, and we have

### Proposition 6

The instances $(M,\omega ,k,d)$ and $(\widetilde{M},\omega ,k,d)$ of Weighted Diverse Bases are equivalent.

Recall the sets $L = \widehat{B} \subseteq S^{*}$ and $L^{*} = (S^{*} {\setminus } L)$ from the construction. $(L,L^*)$ is thus a partition of $E(\widetilde{M}) = S^{*}$. From the construction we get $L = \widehat{B} \subseteq \overline{B}$ and $L^{*} \subseteq B$. Now since $\textsf {rank}(\overline{B})\le \lceil \frac{d}{2}\rceil k$ and $\textsf {corank}(B)\le \lceil \frac{d}{2}\rceil k$ in *M*, we have that for every basis $B'$ of $\widetilde{M}$, $|B'\cap L|\le \lceil \frac{d}{2}\rceil k$ and $|L^* {\setminus } B'|\le \lceil \frac{d}{2}\rceil k$ hold.

This completes the description of the algorithm that returns the instance $(\widetilde{M},\omega ,k,d)$ and the partition $(L,L^*)$ of $E(\widetilde{M})$. Since $|L| \le \lceil \frac{d}{2}\rceil ^2k^3$ and $|L^{*}| \le \lceil \frac{d}{2}\rceil ^2k^3$, we have that $|E(\widetilde{M})|\le 2\lceil \frac{d}{2}\rceil ^2k^3$. It is straightforward to verify that given an independence oracle for *M* we can construct the following in polynomial time: (i) the set $E(\widetilde{M})$, (ii) an independence oracle for $\widetilde{M}$ that in time polynomial in |*E*(*M*)| answers queries for $\widetilde{M}$, and (iii) the sets *L* and $L^*$. To see this, note that $A\subseteq E(\widetilde{M})$ is independent in $\widetilde{M}$ if and only if $A'=A\cup (B\setminus S^*)$ is independent in *M*.

To show the second claim of the lemma, assume that we are given representation ${\textbf {A}}$ of *M* over a finite field $\mathbb {F}$. It is well-known that $M^*$ also is representable over $\mathbb {F}$ and, given ${\textbf {A}}$, the representation of $M^*$ over $\mathcal {F}$ can be computed in polynomial time by linear algebra tools (see, e.g., [22]). Taking into account that contraction of a set is equivalent to the deletion of the same set in the dual matroid and vice versa, we obtain that the representation $\tilde{{\textbf {A}}}$ of $\widetilde{M}$ can be constructed in polynomial time from *A*. This concludes the proof of the lemma. $\square$

Using Lemma 2 we can prove that Weighted Diverse Bases is ${{\,\mathrm{\textsf{FPT}}\,}}$ when parameterized by *k* and *d*.

**Theorem 2** Weighted Diverse Bases *can be solved in* $2^{\mathcal {O}(dk^2(\log k+\log d))}\cdot |E(M)|^{\mathcal {O}(1)}$ *time.*

### Proof

Let $(M,\omega ,k,d)$ be an instance of Weighted Diverse Bases. We run the algorithm from Lemma 2. If the algorithm solves the problem, then we are done. Otherwise, the algorithm outputs an equivalent instance $(\widetilde{M},\omega ,k,d)$ of Weighted Diverse Bases such that $E(\widetilde{M})\subseteq E(M)$ and $|E(\widetilde{M})|\le 2\lceil \frac{d}{2}\rceil ^2k^3$. Moreover, the algorithm computes the partition $(L,L^*)$ of $E(\widetilde{M})$ with the property that for every basis *B* of $\widetilde{M}$, $|B\cap L|\le \lceil \frac{d}{2}\rceil k$ and $|L^*{\setminus } B|\le \lceil \frac{d}{2}\rceil k$. Then we check all possible *k*-tuples of bases by brute force and verify whether there are *k* bases forming a solution. By the properties of *L* and $L^*$, $\widetilde{M}$ has $(d^2k^3)^{\mathcal {O}(dk)}$ distinct bases. Therefore, we check at most $(d^2k^3)^{\mathcal {O}(dk^2)}$ *k*-tuples of bases. We conclude that this checking can be done in $2^{\mathcal {O}(dk^2(\log k+\log d))}\cdot |E(M)|^{\mathcal {O}(1)}$ time, and the claim follows.$\square$

If the input matroid is given by a representation over a finite field, then Weighted Diverse Bases admits a polynomial kernel when parameterized by *k* and *d*.

**Theorem 3** *Given a representation of the matroid* *M* *over a finite field* GF(q) *as input, we can compute a kernel of* Weighted Diverse Bases *of size* $\mathcal {O}(k^6d^4\log q)$.

### Proof

Let $(M,\omega ,k,d)$ be an instance of Weighted Diverse Bases. Let also ${\textbf {A}}$ be its representation over $\textsf {GF(}q{\textsf {)}}$. We run the algorithm from Lemma 2. If the algorithm solves the problem and reports that $(M,\omega ,k,d)$ is a yes-instance, we return a trivial yes-instance of the problem. Otherwise, the algorithm outputs an equivalent instance $(\widetilde{M},\omega ,k,d)$ of Weighted Diverse Bases such that $E(\widetilde{M})\subseteq E(M)$ and $|E(\widetilde{M})|\le 2\lceil \frac{d}{2}\rceil ^2k^3$. Moreover, the algorithm computes a representation $\tilde{{\textbf {A}}}$ of $\widetilde{M}$ over $\textsf {GF(}q{\textsf {)}}$. Clearly, it can be assumed that the number of rows of the matrix $\tilde{{\textbf {A}}}$ equals $\textsf {rank}(\widetilde{M})$. Since $\textsf {rank}(\widetilde{M})\le |E(\widetilde{M})|$, the matrix $\tilde{{\textbf {A}}}$ has $\mathcal {O}(k^6d^4)$ elements. Because $\tilde{{\textbf {A}}}$ is a matrix over $\textsf {GF(}q{\textsf {)}}$, it can be encoded by $\mathcal {O}(k^6d^4\log q)$ bits. Finally, note that the weights of the elements can be truncated by *d*, that is, we can set $\omega (e):=\min \{\omega (e),d\}$ for every $e\in E(\widetilde{M})$. Then the weights can be encoded using $\mathcal {O}(d^2k^3\log d)$ bits. This concludes the construction of our kernel.$\square$

## An ${{\,\mathrm{\textsf{FPT}}\,}}$ algorithm for Weighted Diverse Common Independent Sets

In this section we show that Weighted Diverse Common Independent Sets is ${{\,\mathrm{\textsf{FPT}}\,}}$ when parameterized by *k* and *d*.

We use a similar win-win approach as for Weighted Diverse Bases and observe that if the two matroids from an instance of Weighted Diverse Common Independent Sets have a sufficiently big common independent set, then we have a yes-instance of Weighted Diverse Common Independent Sets.

### Lemma 3

Let $M_1$ and $M_2$ be matroids with a common ground set *E*, and let $k\ge 1$ and $d\ge 1$ be integers. If there is an $X\subseteq E$ of size at least $k\lceil \frac{d}{2}\rceil$ such that *X* is a common independent set of $M_1$ and $M_2$, then $(M_1,M_2,\omega ,k,d)$ is a yes-instance of Weighted Diverse Common Independent Sets for any weight function $\omega :E\rightarrow \mathbb {N}$.

### Proof

Let $X\subseteq E$ be a set of size at least $k\lceil \frac{d}{2}\rceil$ such that *X* is a common independent set of $M_1$ and $M_2$. Then there is a partition $I_1,\ldots ,I_k$ of *X* such that $|I_i|\ge \lceil \frac{d}{2}\rceil$ for every $i\in \{1,\ldots ,k\}$. Clearly, $I_1,\ldots ,I_k$ are common independent sets of $M_1$ and $M_2$. Also we have that $\omega (I_i\bigtriangleup I_j)=\omega (I_i)+\omega (I_j)\ge d$ for all distinct $i,j\in \{1,\ldots ,k \}$ and every weight function $\omega$ which assigns positive integral weights. This means that $I_1,\ldots ,I_k$ is a solution for $(M_1,M_2,\omega ,k,d)$; that is, $(M_1,M_2,\omega ,k,d)$ is a yes-instance.$\square$

Lemma 3 implies that we can assume that the maximum size of a common independent set of the input matroids is bounded. We prove the following crucial lemma.

### Lemma 4

Let $(M_1,M_2,\omega ,k,d)$ be an instance of Weighted Diverse Common Independent Sets such that the maximum size of a common independent set of $M_1$ and $M_2$ is at most *s*. Then there is a set $\mathcal {F}$ of common independent sets of $M_1$ and $M_2$, of size $|\mathcal {F}| = 2^{\mathcal {O}(s^2\log (ks))}\cdot d$, such that if $(M_1,M_2,\omega ,k,d)$ is a yes-instance of Weighted Diverse Common Independent Sets then the instance has a solution $I_1,\ldots ,I_k$ with $I_i\in \mathcal {F}$ for $i\in \{1,\ldots ,k\}$. Moreover, $\mathcal {F}$ can be constructed in $2^{\mathcal {O}(s^2\log (ks))}\cdot d\cdot |E|^{\mathcal {O}(1)}$ time where *E* is the (common) ground set of $M_{1}$ and $M_{2}$.

### Proof

Consider $(M_1,M_2,\omega ,k,d)$. Let $E=E(M_1)= E(M_2)$. It is convenient to assume that the weights of the elements are bounded by *d*. For this, we set $\omega (e):=\min \{d,\omega (e)\}$ for every $e\in E$. It is straightforward to see that by this operation we obtain an equivalent instance of Weighted Diverse Common Independent Sets. Notice that for every common independent set *I* of $M_1,M_2$, we now have $\omega (I)\le ds$.

For every $w\in \{0,\ldots ,ds\}$, we use a recursive branching algorithm to construct a family $\mathcal {F}_w$ of size $2^{\mathcal {O}(s^2\log (ks))}$ of common independent sets of $M_1$ and $M_2$ with the following properties: (i) each set in $\mathcal {F}_w$ has weight *at least* *w*, and (ii) if $S=\{I_1,\ldots ,I_k\}$ is a solution to the instance $(M_1,M_2,\omega ,k,d)$ such that $\omega (I_i)=w$ for some $i\in \{1,\ldots ,k\}$, then there is an $I_i'\in \mathcal {F}_w$ such that $(S {\setminus } \{I_{i}\}) \cup \{I_i'\}$ is also a solution to $(M_1,M_2,\omega ,k,d)$.

The algorithm, denoted by $\mathcal {A}$, takes as its input a common independent set *X* of $M_1$ and $M_2$, and two matroids $M_1'$ and $M_2'$ such that $M_i'=(M_i-W)/X$ for $i=1,2$ for some subset $W\subseteq E{\setminus } X$. For the very first call to $\mathcal {A}$ we set $X:=\emptyset$ and $M_i'=M_i$ for $i=1,2$ (thus we implicitly set $W:=\emptyset$). Algorithm $\mathcal {A}$ outputs at most *ks* common independent sets of $M_1$ and $M_2$ of the form $X \cup Y$, where $Y\subseteq E'=E(M_1')=E(M_2')$ is a common independent set of $M_1'$ and $M_2'$. Note that $E'=E{\setminus } (X \cup W)$. Algorithm $\mathcal {A}$ performs the following steps.

1. If $\omega (X)\ge w$, then output *X* and return.
2. Greedily compute at most *ks* disjoint common independent sets $Y_1,\ldots ,Y_\ell$ of $M_1'$ and $M_2'$, each of weight at least $w'=w-\omega (X)$, as follows.
  - Set $$i = 1, Ys = \{\}$$.
  - If $| Ys | = (i - 1) = ks$ then set $\ell = (i - 1)$ and go to **Step 3**.
  - Set $$M_h''=M_h'- (\displaystyle \bigcup _{Y_{j} \in Ys }Y_{j})$$, for $$h=1,2$$.
  - Find a common independent set *Z* of $M_1''$ and $M_2''$ of the maximum weight.
  - If $\omega (Z)<w'$, then set $\ell = (i - 1)$ and go to **Step 3**. Otherwise, set $Y_{i} = Z, Ys = ( Ys \cup \{Y_{i}\})$ and $i = i + 1$, and go to **Step 2(b)**.
3. At this point we have $Ys = \{Y_1,\ldots ,Y_\ell \}$.
  If $\ell =0$ then return.
  If $\ell =ks$ then output the sets $X\cup Y_1,\ldots ,X\cup Y_\ell$ and return.
  If neither of the above holds then:
    Set $$R=\displaystyle \bigcup _{Y_{j} \in Ys }Y_{j}$$.
    For each nonempty common independent set $Z\subseteq R$ of $M_1'$ and $M_2'$, set $W=R\setminus Z$ and recursively invoke $\mathcal {A}(X\cup Z,(M_1'-W)/Z,(M_2'-W)/Z)$.

This completes the description of $\mathcal {A}$. To construct $\mathcal {F}_w$, we call $\mathcal {A}(\emptyset ,M_1,M_2)$. Then the set $\mathcal {F}_w$ includes all the sets output by $\mathcal {A}$. Note that in every recursive step we call $\mathcal {A}(X\cup Z,(M_1'-W)/Z,(M_2'-W)/Z)$ only if $Z\ne \emptyset$. So the size of the first argument $(X\cup Z)$ to a recursive call of $\mathcal {A}$ is strictly larger than the size of the first argument *X* of the parent call to $\mathcal {A}$. Moreover, since *Z* is a common independent set of $M_1'$ and $M_2'$, we have that $X\cup Z$ is a common independent set of $M_1$ and $M_2$. Because the maximum size of the common independent set of $M_1$ and $M_2$ is at most *s*, we obtain that the depth of the recursion is bounded by *s*, that is, the algorithm is finite. We show the crucial property of $\mathcal {F}_w$ mentioned above.

### Proposition 7

If $S=\{I_1,\ldots ,I_k\}$ is a solution to the instance $(M_1,M_2,\omega ,k,d)$ such that $\omega (I_i)=w$ for some $i\in \{1,\ldots ,k\}$, then there is an $I_i'\in \mathcal {F}_w$ such that $(S {\setminus } \{I_{i}\}) \cup \{I_i'\}$ is also a solution to $(M_1,M_2,\omega ,k,d)$.

### Proof

Fix a set $I_{i} \in S;\;\omega (I_{i}) = w$. Recall that an arbitrary invocation of $\mathcal {A}$ has the form $\mathcal {A}(X, (M_{1} - W)/X, (M_{2} - W)/X)$ where *X* is a common independent set of $M_{1}, M_{2}$ and $W \subseteq (E \setminus X)$. For the very first invocation of $\mathcal {A}$ these sets are $X = \emptyset , W = \emptyset$, and these sets trivially satisfy the **viability condition** $(X \cup W) \cap I_{i} = X$; that is: $I_{i}$ *contains all of* *X*, *and none of* *W*. We show that any invocation of $\mathcal {A}$ whose arguments satisfy the viability condition *either* outputs a set $I_i'$ that can be used to replace $I_i$ in *S*, *or* makes at least one recursive call to $\mathcal {A}$ such that the arguments to this recursive call satisfy the viability condition. Since the size of the first argument $(X\cup Z)$ to a recursive call of $\mathcal {A}$ is strictly larger than the size of the first argument *X* of the parent call to $\mathcal {A}$, we get that some call to $\mathcal {A}$ will output a set $I_{i}'$ with the desired property.

Assume inductively that $\mathcal {A}(X, M_{1}' = (M_{1} - W)/X, M_{2}' = (M_{2} - W)/X)$ is an invocation of $\mathcal {A}$ whose arguments satisfy the viability condition. If $\omega (X)\ge w$, then $X=I_i$, because $\omega (I_i)=w$ and the weights are non-negative. In this case, the algorithm outputs $X=I_i$ in **Step 1**. Clearly, we can set $I_i'=I_i$, and we are done. So let us assume that this is not the case, and that $\omega (X)<w$. In this case $X \subsetneq I_{i}$ holds and the algorithm goes to **Step 2**.

Let $E' = E \setminus (X \cup W)$ be the common ground set of $M_{1}'$ and $M_{2}'$. Let $Y = (I_{i} {\setminus } X)$ and $w'=\omega (Y)=w-\omega (X)$. Then since *Y* is a common independent set of $M_{1}'$ and $M_{2}'$, the greedy computation of **Step 2** produces a *nonempty* family $Y_1,\ldots ,Y_\ell$ of disjoint common independent sets of $M_1'$ and $M_2'$ of weight at least $w'$ each. Note that $X\cup Y_1,\ldots ,X\cup Y_\ell$ are common independent sets of $M_1$ and $M_2$. We consider two cases depending on the value of $\ell$ in **Step 3**. Note that by the above reasoning the case $\ell = 0$ does not arise here.

1. $\ell =ks$. In this case the algorithm outputs the sets $X\cup Y_1,\ldots ,X\cup Y_\ell$ where $(X \cap Y_{i}) = \emptyset$ holds for all $i\in \{1,\dotsc ,\ell \}$. Recall that every common independent set of $M_1$ and $M_2$ has size at most *s*. Therefore, the set $J=\bigcup _{j\in \{1,\ldots ,k\},~j\ne i}I_j$ has size at most $(k-1)s<\ell$. Hence, by the pigeonhole principle, there is an $h\in \{1,\ldots ,\ell \}$ such that $Y_h\cap I_j=\emptyset$ holds for all $j\in \{1,\ldots ,k\}\;;\;j\ne i$.
  Let $I_i':=X\cup Y_h$. Then $I_i'$ is a common independent set of $M_1$ and $M_2$ and $\omega (I_i') = \omega (X)+\omega (Y_h) \ge \omega (X)+w' = \omega (X)+\omega (I_{i} {\setminus } X) = \omega (I_{i})$, where the last equation follows from the fact that $X\subseteq I_{i}$ holds. From this chain of relations we also get that $\omega (I_{i} {\setminus } X) \le \omega (Y_h)$ holds. Consider an arbitrary index $j\in \{1,\ldots ,k\}\;;\;j\ne i$. Then $\omega (I_{i} \bigtriangleup I_{j}) = \omega (I_{j} {\setminus } I_{i}) + \omega (I_{i} {\setminus } I_{j}) \le \omega (I_{j} {\setminus } X) + \omega (I_{i} {\setminus } I_{j}) = \omega (I_{j} {\setminus } X) + \omega (X {\setminus } I_{j}) + \omega ((I_{i} {\setminus } X) {\setminus } I_{j}) \le \omega (I_{j} \bigtriangleup X) + \omega (I_{i} {\setminus } X) \le \omega (I_{j} \bigtriangleup X) + \omega (Y_{h})$. But since $I_i'=X\cup Y_h$, $(X \cap Y_{h}) = \emptyset$ and $(I_{j} \cap Y_{h}) = \emptyset$ we get that $\omega (I_{j} \bigtriangleup I_{i'}) = \omega (I_{j} \bigtriangleup X) + \omega (Y_{h})$ holds. Thus $\omega (I_{j} \bigtriangleup I_{i'}) \ge \omega (I_{i} \bigtriangleup I_{j})$, and so replacing $I_i$ by $I_i'$ in the solution *S* indeed gives us a solution to the instance $(M_1,M_2,\omega ,k,d)$.
2. $0< \ell < ks$. In this case we set $R:=\bigcup _{i=1}^{\ell }Y_i$. From the construction we get that the matroids $M_1'-R$ and $M_2'-R$ have no common independent set of weight at least $w'$. Since $(I_{1} {\setminus } X) {\setminus } R$ is such a common independent set we have that $\omega ((I_{1} {\setminus } X) {\setminus } R) < w'$, and since $\omega (I_{i}{\setminus } X)=w'$ we get that $Z=(I_i{\setminus } X)\cap R\ne \emptyset$. Clearly, *Z* is a common independent set of $M_1'$ and $M_2'$. Our algorithm considers all such sets. Hence there is a recursive call $\mathcal {A}(X',(M_1'-W)/Z,(M_2'-W)/Z)$ where $Z = ((I_i{\setminus } X)\cap R), X' = X \cup Z, W=R{\setminus } Z$. By the choice of *Z* and *W* we get that $(X' \cup W) \cap I_{i} = X'$, so that this recursive call satisfies the viability condition. Moreover, we have that $|X'|>|X|$. This completes the second case and the proof of the proposition.

$$\square $$

We already observed that the algorithm $\mathcal {A}$ is finite. Now we evaluate its running time and the size of $\mathcal {F}_w$.

### Proposition 8

The set $\mathcal {F}_w$ has size $2^{\mathcal {O}(s^2\log (ks))}$ and can be constructed in $2^{\mathcal {O}(s^2\log (ks))}\cdot |E|^{\mathcal {O}(1)}$ time.

### Proof

To give an upper bound on the size of $\mathcal {F}_w$, observe that in each recursive call, the algorithm $\mathcal {A}$ either outputs some sets, or performs some recursive calls, or simply returns without outputting anything. Notice that in Step 1, $\mathcal {A}$ can output at most one set, and $\mathcal {A}$ may output *ks* sets in Step 3. The number of recursive calls is upper bounded by the number of nonempty common independent sets $Z\subseteq R$ of $M_1'$ and $M_2'$. Since $\ell <ks$ and $|Y_i|\le s$ for $i\in \{1,\ldots ,\ell \}$, $|R|\le ks^2$. Because for each *Z*, $|Z|\le s$, the branching factor is at most $(ks^2)^s=2^{\mathcal {O}(s\log (ks))}$. Since the depth of the recursion is at most *s*, the search tree has $2^{\mathcal {O}(s^2\log (ks))}$ leaves. This implies that the size of $\mathcal {F}_w$ is $2^{\mathcal {O}(s^2\log (ks))}$.

To evaluate the running time, note that in Step 2, the algorithm greedily constructs the sets $Y_1,\ldots ,Y_\ell$ that are common independent sets of $M_1'$ and $M_2'$. By Proposition 1, this can be done in polynomial time, because in each iteration we find a common independent set of maximum weight. Because the search tree has $2^{\mathcal {O}(s^2\log (ks))}$ leaves, the total running time is $2^{\mathcal {O}(s^2\log (ks))}\cdot |E|^{\mathcal {O}(1)}$.$\square$

We construct $\mathcal {F}=\bigcup _{w=0}^{ds}\mathcal {F}_w$. By Proposition 8, $|\mathcal {F}|\le (ds+1)\max _{w\in \{0,\ldots ,ds\}}|\mathcal {F}_{w}|=2^{\mathcal {O}(s^2\log (ks))}\cdot d$ and $\mathcal {F}$ can be constructed in total $2^{\mathcal {O}(s^2\log (ks))}\cdot d\cdot |E|^{\mathcal {O}(1)}$ time. Proposition 7 implies that if $(M_1,M_2,\omega ,k,d)$ is a yes-instance of Weighted Diverse Common Independent Sets, then the instance has a solution $I_1,\ldots ,I_k$ with $I_i\in \mathcal {F}$ for $i\in \{1,\ldots ,n\}$. $\square$

Combining Lemma 3 and Lemma 4, we obtain the main result of the section.

**Theorem 4** Weighted Diverse Common Independent Sets can be solved in $2^{\mathcal {O}(k^3d^2\log (kd))}\cdot |E|^{\mathcal {O}(1)}$ time.

### Proof

Let $(M_1,M_2,\omega ,k,d)$ be an instance of Weighted Diverse Common Independent Sets. First, we use Proposition 1 to solve Matroid Intersection for $M_1$ and $M_2$ and find a common independent set *X* of maximum size. If $|X|\ge k\lceil \frac{d}{2}\rceil$, then by Lemma 3, we conclude that $(M_1,M_2,\omega ,k,d)$ is a yes-instance. Assume that this is not the case. Then the maximum size of a common independent set of $M_1$ and $M_2$ is $s< k\lceil \frac{d}{2}\rceil$. We apply Lemma 4 and construct the set $\mathcal {F}$ of size $2^{\mathcal {O}((kd)^2\log (kd))}$ in $2^{\mathcal {O}((kd)^2\log (kd))}\cdot |E|^{\mathcal {O}(1)}$ time. By this lemma, if $(M_1,M_2,\omega ,k,d)$ is a yes-instance, it has a solution $I_1,\ldots ,I_k$ such that $I_i\in \mathcal {F}$ for $i\in \{1,\ldots ,k\}$. Hence, to solve the problem we go over all *k*-tuples of the elements of $\mathcal {F}$, and for each *k*-tuple, we verify whether these common independent sets of $M_1$ and $M_2$ give a solution. Clearly, we have to consider $2^{\mathcal {O}(k^3d^2\log (kd))}$ tuples. Hence, the total running time is $2^{\mathcal {O}(k^3d^2\log (kd))}\cdot |E|^{\mathcal {O}(1)}$. $\square$

## Perfect Matchings

In this section we prove that Diverse Perfect Matchings is fixed parameter tractable when parameterized by *k* and *d*. We need the following simple observations later in this section.

### Observation 3

The cardinality of symmetric differences of perfect matchings in a graph obeys the triangle inequality. That is, for a graph *G* and perfect matchings $M_1,M_2,M_3$ in *G*, $\vert M_1\bigtriangleup M_2\vert + \vert M_2\bigtriangleup M_3\vert \ge \vert M_1\bigtriangleup M_3\vert$.

Observation 3 follows from the fact that Hamming distance is a metric and hence obeys triangular inequality.

### Observation 4

Let *G* be a graph and $M_1$ and $M_2$ be two perfect matchings in *G*. Then $\vert M_1\bigtriangleup M_2\vert =2\cdot \vert M_1{\setminus } M_2\vert =2\cdot \vert M_2{\setminus } M_1\vert$.

For an undirected graph *G*, the Tutte matrix ${\textbf {A}}$ of *G* over the field ${{\mathbb {F}}}_2[X]$ is defined as follows, where ${\mathbb F}_2$ is the Galois field on $\{0,1\}$ and $X=\{x_e ~:~ e\in E(G)\}$. The rows and columns of ${\textbf {A}}$ are labeled with *V*(*G*) and for each $e=\{u,v\}\in E(G)$, ${\textbf {A}} [u,v]={\textbf {A}} [v,u]=x_e$. All other entries in the matrix are zeros. That is, for any pair of vertices $u,v\in V(G)$, if there is no edge between *u* and *v*, ${\textbf {A}} [u,v]=0$. It is well known that $det(A)\ne 0$ if and only if *G* has a perfect matching. As the characterstic of ${{\mathbb {F}}}_2$ is a 2, the determinant of ${\textbf {A}}$ coincides with the permanent of ${\textbf {A}}$. That is,

1 $$\begin{aligned} det({\textbf {A}})=perm({\textbf {A}})=\sum _{\sigma \in S_{V(G)}} \Pi _{v\in V(G)} {\textbf {A}} [v,\sigma (v)]. \end{aligned}$$

Here, $S_{V(G)}$ is the set of all permutations of *V*(*G*). Let $\textsf{PM}(G)$ be the set of perfect matchings on *G*. Then, one can show that $det({\textbf {A}})=\sum _{M\in \textsf{PM}(G)} \Pi _{e\in M} x_e^2$.

Let *Y* be a set of variables disjoint from *X*. For each edge *e*, let $L(e)\subseteq Y$ be a subset of variables. Let ${\textbf {A}} '$ be the matrix obtained from ${\textbf {A}}$ by replacing each entry of the form $x_e$ with $x_e\cdot \Pi _{y\in L(e)}y$. Then,

2 $$\begin{aligned} det({\textbf {A}} ')=perm({\textbf {A}} ')= & {} \sum _{\sigma \in S_{V(G)}} \Pi _{v\in V(G)} {\textbf {A}} '[v,\sigma (v)] \nonumber \\= & {} \sum _{\sigma \in S_{V(G)}} \Pi _{v\in V(G)} \left( {\textbf {A}} [v,\sigma (v)] \cdot \Pi _{y\in L(\{v,\sigma (v)\})}y\right) , \end{aligned}$$

where $\Pi _{y\in L(\{v,\sigma (v)\})}y=1$ if $\{v,\sigma (v)\}\notin E(G)$ or $L(e)=\emptyset$.

### Lemma 5

Let *G* be an undirected graph and let $X=\{x_e ~:~e\in E(G)\}$ and $Y=\{y_1,\ldots ,y_{\ell }\}$ be two sets of variables such that $X\cap Y=\emptyset$. For each edge $e\in E(G)$, we are also given a subset $L(e)\subseteq Y$. Let ${\textbf {A}} '$ be the matrix defined as above. For any perfect matching *M*, $\Pi _{e\in M} x_e^2 \Pi _{y\in L(e)}y^2$ is a monomial in $det({\textbf {A}} ')$. Moreover, for any monomial *m* in $det({\textbf {A}} ')$, $M'=\{e ~:~ x_e \text{ is } \text{ a } \text{ variable } \text{ in } m\}$ is a perfect matching in *G* and for each $e\in M'$, *L*(*e*) is a subset of variables in the monomial *m*.

### Proof

A *cycle-matching cover* of *G* is a subset of edges $F\subseteq E(G)$ such that $V(F)=V(G)$ and each connected component of *G*[*F*] is either a cycle or an edge. For each non-zero term in the summation of (2), there is a cycle-matching cover defined as follows. Let $\sigma \in S_{V(G)}$ such that $\Pi _{v\in V(G)} {\textbf {A}} [v,\sigma (v)] \cdot \Pi _{y\in L(\{v,\sigma (v)\})}y$ is non-zero. Then, $\Pi _{v\in V(G)} {\textbf {A}} [v,\sigma (v)]$ is non-zero. As *G* is a simple graph, $A[v,v]=0$. Therefore, since $\Pi _{v\in V(G)} {\textbf {A}} [v,\sigma (v)] \ne 0$, there is no 1-cycle in $\sigma$. Moreover any $\ell$-cycle in $\sigma$ corresponds to a cycle in *G* and any 2-cycle in $\sigma$ corresponds to an edge in *G*, where the vertices covered in the cycle are the vertices present the cycle of the permutation. That is, for each cycle $(u_1,u_2,\ldots ,u_{\ell }$ in $\sigma$, $u_1,u_2,\ldots ,u_{\ell },u_1$ is a cycle in *G* if $\ell >1$ and $u_1u_2$ is a matching edge if $\ell =2$. Therefore, there is a cycle-matching cover corresponding to the non-zero term $\Pi _{v\in V(G)} {\textbf {A}} [v,\sigma (v)] \cdot \Pi _{y\in L(\{v,\sigma (v)\})}y$.

Let *F* be a cycle-matching cover. Let $\{C_1,\ldots , C_r\}$ be the set of cycles in *G*[*F*] and $\{e_1,\ldots ,e_{s}\}$ be the set of the edges in $F{\setminus } (\bigcup _{i} E(C_i))$. Let $F'=\bigcup _{i\in [r]} E(C_i)$. For each cycle $C=u_1,u_2,\ldots ,u_{\ell },u_1$ in *G*[*F*], where $\ell >2$ one can define two permutations $\sigma _1$ and $\sigma _2$ on *V*(*C*) as follows: $(u_1,u_2,\ldots ,u_{\ell })$ and $(u_1,u_{\ell },u_{\ell -1},\ldots ,u_{2})$. That is,

$$\begin{aligned}\Pi _{v\in V(C)} {\textbf {A}} [v,\sigma _1(v)]=\Pi _{v\in V(C)} {\textbf {A}} [v,\sigma _2(v)]=\Pi _{e\in E(C)}\left( x_e\cdot \Pi _{y\in L(e)}y\right) .\end{aligned}$$

This implies that there are $2^{r}$ terms in (2) which are equal to $\sum _{e\in F{\setminus } F'}\left( x_e\cdot \Pi _{y\in L(e)}y\right) ^2 + \sum _{e\in F'}\Pi _{e\in F'}\left( x_e\cdot \Pi _{y\in L(e)}y\right)$ (which we call the terms corresponding to *F*). In other words if *F* is a perfect matching then $\Pi _{e\in F}\left( x_e\cdot \Pi _{y\in L(e)}y\right) ^2$ is a unique term in (2), and if *F* is has cycle then the terms corresponding to *F* will cancel each other, because the characteristic of the ${{\mathbb {F}}}_2[X\cup Y]$ is 2. Therefore, for any perfect matching *M*, $\Pi _{e\in M} x_e^2 \Pi _{y\in L(e)}y^2$ is a monomial in $det({\textbf {A}} ')$.

As any non-zero term in (2) corresponds to a cycle-matching cover, and for any cycle matching cover that contains at least one cycle all the terms corresponding to it cancels each other we have the following. For any monomial *m* in $det({\textbf {A}} ')$, $M'=\{e ~:~ x_e \text{ is } \text{ a } \text{ variable } \text{ in } m\}$ is perfect a matching in *G*. Also, from the construction of ${\textbf {A}} '$, it follows that for each $e\in M'$, *L*(*e*) is subset of variables in *m*.$\square$

We use the following two known results.

### Proposition 9

(Schwartz-Zippel Lemma [24, 28]) Let $P(x_1, \ldots , x_n)$ be a multivariate polynomial of total degree at most *d* over a field ${{\mathbb {F}}}$, and *P* is not identically zero. Let $r_1,\ldots ,r_n$ be the elements in ${{\mathbb {F}}}$ chosen uniformly at random with repetition. Then ${{\text {Pr}}}(P(r_1,..., r_n) = 0) \le \frac{d}{|{\mathbb F}|}$.

For a multivariate polynomial *P* and a monomial *m*, we let *P*(*m*) denote the coefficient of *m* in *P*.

### Proposition 10

( [27]) Let $P(x_1, \ldots , x_n)$ be a polynomial over a field of characteristic two, and $T \subseteq [n]$ be a set of target indices. For a set $I \subseteq [n]$, define $P_{-I} (x_1, \ldots , x_n) = P(y_1, \ldots , y_n)$ where $y_i = 0$ for $i \in I$ and $y_i = x_i$ otherwise. Define

$$\begin{aligned} Q(x_1,\ldots , x_n) = \sum _{I\subseteq T} P_{-I} (x_1, \ldots , x_n). \end{aligned}$$

Then, for any monomial m such that $t:= \Pi _{i\in T}x_i$ divides *m* we have $Q(m) = P(m)$, and for every other monomial we have $Q(m) = 0$.

### Lemma 6

There is an algorithm that given an undirected graph *G*, perfect matchings $M_1,\ldots , M_r$, and a non-negative integer *s*, runs in time $2^{\mathcal {O}(rs)} n^{\mathcal {O}(1)}$, and outputs a perfect matching *M* such that $\vert M \setminus M_i\vert \ge s$ for all $i\in \{1,\ldots ,r\}$ (if such a matching exists) with probability at least $\frac{2}{3}e^{-rs}$.

### Proof

For each $i\in \{1,\ldots ,r\}$, we color each edge in $E(G){\setminus } M_i$ uniformly at random using colors $\{c_{i,1},\ldots ,c_{i,s}\}$. Now we label each edge with a subset of the variable set $Y=\{y_{i,j}~:~ i\in [r], j\in [s]\}$. For each edge *e*, we label it with

$$\begin{aligned} L(e)=\{y_{i,j}~:~ i\in [r] \text{ and } \text{ e } \text{ is } \text{ colored } \text{ with } c_{i,j} \text{ in } \text{ the } \text{ random } \text{ coloring } \text{ for } \text{ i }\}. \end{aligned}$$

Let ${\textbf {A}}$ be the Tutte matrix of *G* over the field ${{\mathbb {F}}}_2[X]$, where $X=\{x_e :e\in E(G)\}$. Let ${\textbf {A}} '$ be the matrix obtained from ${\textbf {A}}$ by replacing each entry of the form $x_e$ with $x_e\cdot \Pi _{y\in L(e)}y$.

Suppose there is a matching *M* such that $\vert M {\setminus } M_i\vert \ge s$ for all $i\in \{1,\ldots ,r\}$. Then, for each $i\in [r]$, let $\{e_{i,1},\ldots ,e_{i,s}\}\subseteq (M {\setminus } M_i)$ be an arbitrary subset. We say that $\{e_{i,1},\ldots ,e_{i,s}\}$ is colorful if the edges in $\{e_{i,1},\ldots ,e_{i,s}\}$ gets distinct colors from $\{c_{i,1},\ldots ,c_{i,s}\}$ in the random coloring for *i*. Then for each $i\in [r]$, the probability that $\{e_{i,1},\ldots ,e_{i,s}\}$ is colorful is $\frac{s!}{s^s}\ge e^{-s}$. For each $q\in [r]$, let $E_q$ be the event that $\{e_{q,1},\ldots ,e_{q,s}\}$ is colorful. As the random coloring for $i\in [r]$ is different from the random coloring for $j\in [r]{\setminus } \{i\}$, the events $E_i$ and $E_j$ are independent. That is, $E_1,\ldots ,E_r$ are independent events and hence ${{\text {Pr}}}[\bigcap _{i=1}^r E_i]\ge e^{-rs}$. Therefore, there is a monomial *m* in $det({\textbf {A}} ')$ with probability at least $e^{-rs}$ such that $M=\{e\in E(G) ~:~ x_e \text{ is } \text{ a } \text{ variable } \text{ in } m\}$ and *Y* is a subset of variables in *m*.

Now, suppose there is a monomial *m* in $det({\textbf {A}} ')$ such that *Y* is a subset of variables in *m*. Therefore, since for each $i\in [r]$ only the edges in $E(G)\setminus M_i$ are colored and $\{y_{i,j}~:~j\in [s]\}\subseteq Y$, we have that $\vert M\setminus M_i\vert \ge s$. Moreover, by Lemma 5, $\{e \in E(G)~:~ x_e \text{ is } \text{ a } \text{ variable } \text{ in } m\}$ is a perfect matching in *G*.

Thus, it is enough to check whether there exists a monomial *m* in $det({\textbf {A}} ')$ such that all the variables in *Y* are present in *m*.

### Proposition 11

There is an algorithm, that runs in time $2^{\mathcal {O}(|Y|)} n^{\mathcal {O}(1)}$ and it outputs the following. If there is no monomial in $det({\textbf {A}} ')$ that contains *Y*, then the algorithm outputs No. If there is a monomial in $det({\textbf {A}} ')$ that contains *Y*, then the algorithm outputs $Z\subseteq X$ with probability at least 2/3 such that there is a monomial *m* in $det({\textbf {A}} ')$ with variables in *m* is exactly equal to $Z\cup Y$.

### Proof

Let $\{e_1,\ldots ,e_{m}\}=E(G)$. Let $P(x_{e_1},\ldots ,x_{e_m},y_{1,1},\ldots ,y_{r,s})=det({\textbf {A}} ')$. For each $I\subseteq Y$, we define $P_{-I}(x_{e_1},\ldots ,x_{e_m},y_{1,1},\ldots ,y_{r,s})=P(x_{e_1},\ldots ,x_{e_m},z_{1,1},\ldots ,z_{r,s})$, where for all $i\in [r]$ and $j\in [s]$, $z_{i,j}=0$ if $y_{i,j}\in I$ and $z_{i,j}=y_{i,j}$ otherwise. Let

$$\begin{aligned} Q=Q(x_{e_1},\ldots ,x_{e_m},y_{1,1},\ldots ,y_{r,s})=\sum _{I\subseteq Y} P_{-I}(x_{e_1},\ldots ,x_{e_m},y_{1,1},\ldots ,y_{r,s}). \end{aligned}$$

By Proposition 10, the set of monomials in *Q* are the set of monomial is $det({\textbf {A}} ')$ that contains *Y*. Our objective is to find out the variables in such a monomial if it exists. Towards that we consider *Q* be a polynomial in a field extension ${{\mathbb {F}}}'$ of ${{\mathbb {F}}_2}$ such that the number of elements in the field ${{\mathbb {F}}}'$ is at least *t*, which we fix later. From the construction of $det({\textbf {A}} ')$, we know that the degree of $det({\textbf {A}} ')$ and *Q* is at most $d=n+2rs$. By Proposition 9, the existence of a required monomial can be tested in polynomial time with failure probability at most $\frac{d}{t}$. But, recall that we need to find out the variables in such a monomial. Towards that we do the following. Notice that if *Q* is a polynomial identically zero, then our answer is No. This can be checked using Proposition 9. Assume that *Q* is not identically zero. Let $Q_1=Q(0,x_{e_2},\ldots ,x_{e_m},y_{1,1},\ldots ,y_{r,s})$. If $Q_1$ is not identically equal to zero, then a monomial satisfying the property mentioned in the proposition is present in $Q_1$. Otherwise we know that every monomial with the required property contains the variable $x_{e_1}$. Again this can be checked using Proposition 9. So if $Q_1$ is not identically zero, then set $Q=Q_1$. Next, we let $Q_2=Q(x_{e_1},0,x_{e_2},\ldots ,x_{e_m},y_{1,1},\ldots ,y_{r,s})$. Again, $Q_2$ is not identically equal to zero, then a monomial satisfying the property mentioned in the proposition, is present in $Q_2$. Otherwise we know that every monomial with the required property contains the variable $x_{e_2}$. By repeating this process at most *m* times, we will be able to obtain all the variables present in the required monomial. Our algorithm will succeed if all the $m+1$ application of Proposition 9 do not fail. Thus, by union bound the failure probability of is at most $(m+1)\frac{d}{t}$. We set $(m+1)\frac{d}{t}=\frac{1}{3}$ and this implies that $t=(m+1)(n+rs)$. Hence the success probability of our algorithm is at least $\frac{2}{3}$.

Towards the running time analysis, notice that the construction of the polynomial *Q* takes time $2^{\vert Y\vert } n^{\mathcal {O}(1)}$ and each application of Proposition 9 takes time polynomial in the size of *Q*. This implies that the total running time is bounded by $2^{\mathcal {O}(|Y|)} n^{\mathcal {O}(1)}$.$\square$

Now we run the algorithm in Proposition 11, and get a subset $Z\subseteq \{x_e~:~ e\in E(G)\}$ (if it exists) such that there is a monomial *m* in $det({\textbf {A}} ')$ and the variables in *m* is exactly equal to $Z\cup Y$ with probability at least 2/3. As the initial random coloring of edges succeeds with probability at least $e^{-rs}$, the success probability of out algorithm is at least $\frac{2}{3}e^{-rs}$. If no such monomial exists then the algorithm outputs No. By Lemma 5, $M=\{e~:~ x_e\in Z\}$ is a perfect matching and it is the required output. The running time of the algorithm follows from Proposition 11. This completes the proof of the lemma. $\square$

### Lemma 7

There is an algorithm that given an undirected graph *G*, a perfect matching *M*, and non-negative integers *r*, *d*, *s*, runs in time $2^{\mathcal {O}(r^2s)} n^{\mathcal {O}(1)}$, and outputs *r* perfect matchings $M_1^{\star },\ldots ,M_r^{\star }$ such that $\vert M \bigtriangleup M_i^{\star }\vert \le s$ for all $i\in \{1,\ldots ,r\}$ and $\vert M_i^{\star } \bigtriangleup M_j^{\star } \vert \ge d$ for all distinct $i,j\in [r]$ (if such matchings exist) with probability at least $e^{-rs}$. If no such perfect matchings exist, then the algorithm outputs No.

### Proof

Suppose there exist perfect matchings $M_1,\ldots ,M_r$ such that $\vert M \bigtriangleup M_i\vert \le s$ for all $i\in \{1,\ldots ,r\}$ and $\vert M_i \bigtriangleup M_j \vert \ge d$ for all distinct $i,j\in [r]$. Then we know that $\sum _{i=1}^r\vert M \bigtriangleup M_i\vert \le rs$. Let $S_i=M \bigtriangleup M_i$ for all $i\in [r]$. Notice that, as *M* and $M_i$ are perfect matchings $S_i$ forms a collection of alternating cycles (i.e., edges in the cycles alternate between *M* and $M_i$). Let $S=\bigcup _{i=1}^r S_i$.

We do a random coloring on the edges of *G* using *rs* colors. That is, we color each edge of *G* uniformly at random with a color from $\{1,\ldots ,rs\}$. We say that the random coloring is *good* if all the edges in *S* gets distinct colors. The probability that the random coloring is good is $e^{-rs}$.

Now on assume that the random coloring is good. For each $i\in [r]$, let $C_i$ be the set of colors on the edges $S_i$. Notice that $\vert C_i\vert =\vert S_i \vert$.

### Proposition 12

For any two distinct integers $i,j\in [r]$, $\vert C_i\bigtriangleup C_j \vert \ge d$.

### Proof

We know that $\vert M_i \bigtriangleup M_j \vert \ge d$. Let $E_i=M_i{\setminus } M_j$ and $E_j=M_j{\setminus } M_i$. Notice that $\vert E_i\vert + \vert E_j\vert \ge d$ and $E_i\cap E_j=\emptyset$.

Let $E_{i,1}=E_i\cap M$, $E_{i,2}=E_i{\setminus } E_{j,1}$, $E_{j,1}=E_j\cap M$, and $E_{j,2}=E_j{\setminus } E_{j,1}$. As $E_{i,1} \subseteq M{\setminus } M_j$ and $E_{i,1}\subseteq M\cap M_i$, we have that $E_{i,1}\subseteq S_{j}\setminus S_i$. Similarly $E_{j,1}\subseteq S_i\setminus S_j$. As $E_{i,2}\subseteq M_i {\setminus } (M_j\cup M)$, we have that $E_{i,2}\subseteq S_i{\setminus } S_j$. Similarly, we have $E_{j,2}\subseteq S_j{\setminus } S_i$. That is, we prove that $E_{i,1}\cup E_{j,2} \subseteq S_{j}\setminus S_i$ and $E_{j,1}\cup E_{i,2} \subseteq S_{i}\setminus S_j$. Also, since all the edges in *S* gets distinct colors and the colors on the edges in $S_i$ and $S_j$ are $C_i$ and $C_j$, respectively, we have that $\vert C_i\bigtriangleup C_j \vert \ge \vert E_i\cup E_j\vert \ge d$. This completes the proof of the proposition. $\square$

Next we prove the reverse direction of the above claim.

### Proposition 13

Let $Q_1$ and $Q_2$ be two collections of alternating cycles in *G* (i.e., the edges in $Q_i$ are alternating between *M* and $E(G)\setminus M$ for each $i\in \{1,2\}$) such that following hold: $\vert E(Q_1)\vert =\vert C_1\vert$, $\vert E(Q_2)\vert =\vert C_2\vert$, the edges in $E(Q_1)$ uses distinct colors from $C_1$, and the edges in $E(Q_2)$ uses distinct colors from $C_2$. Let $P_1= E(Q_1)\bigtriangleup M$ and $P_2= E(Q_2)\bigtriangleup M$. Then, $P_1$ and $P_2$ are perfect matchings and $\vert P_1\bigtriangleup P_2 \vert \ge d$.

### Proof

As *M* is a perfect matching and $Q_1$ is a collection of alternating cycles, we have that $P_1=E(Q_1)\bigtriangleup M$ is a perfect matching. By similar arguments, we have that $P_2$ is a perfect matching. Now we prove that $E(Q_1)\bigtriangleup E(Q_2)\subseteq P_1 \bigtriangleup P_2$. Consider an edge $e\in E(Q_1)\setminus E(Q_2)$. We have two cases based on whether $e\in M$ or not. In the first case, assume that $e\in M$. Since $e\in E(Q_1)$, $e\in M$, and $P_1=E(Q_1)\bigtriangleup M$, we have that $e\notin P_1$. Also since $e\notin E(Q_2)$, $e\in M$, and $P_2=E(Q_2)\bigtriangleup M$, we have that $e\in P_2$. Therefore, $e\in P_1 \bigtriangleup P_2$.

For the second case, we have that $e\notin M$. Since $e\in E(Q_1)$, $e\notin M$, and $P_1=E(Q_1)\bigtriangleup M$, we have that $e\in P_1$. Also, since $e\notin E(Q_2)$ and $e\notin M$, we have that $e\notin P_2$. Therefore, $e\in P_1\bigtriangleup P_2$.

By arguments, similar to above, one can prove that an edge $e'\in E(Q_2){\setminus } E(Q_1)$ also belongs to $P_1\bigtriangleup P_2$. Thus, we proved that $E(Q_1)\bigtriangleup E(Q_2)\subseteq P_1 \bigtriangleup P_2$. Since $\vert E(Q_1)\vert =\vert C_1\vert$, $\vert E(Q_2)\vert =\vert C_2\vert$, the edges in $E(Q_1)$ uses distinct colors from $C_1$, and the edges in $E(Q_2)$ uses distinct colors from $C_2$, we have that $\vert E(Q_1)\bigtriangleup E(Q_2)\vert \ge \vert C_1\bigtriangleup C_2\vert \ge d$. Therefore, $\vert P_1 \bigtriangleup P_2\vert \ge \vert E(Q_1)\bigtriangleup E(Q_2)\vert \ge d$.$\square$

Thus, to prove the lemma, it is enough to find a collection $Q_i$ of alternating cycles such that $\vert E(Q_i)\vert =\vert C_i\vert$, the edges in $E(Q_i)$ uses distinct colors from $C_i$, for each $i\in [r]$. That is, our algorithm guesses $C_1,\ldots C_r$ and computes $Q_1,\ldots ,Q_r$. The cost of guessing $C_1,\ldots ,C_r$ is $2^{r^2s}$. Now, given $C_i$, to compute $Q_i$ with desired property ($\vert E(Q_i)\vert =\vert C_i\vert$, the edges in $E(Q_i)$ are colored with distinct colors from $C_i$), we design a simple dynamic programming (DP) algorithm. We give a brief outline of this algorithm below. For each subset $L\subseteq C_i$ and pair of vertices *u*, *v* we have table entries *D*[*L*, *u*, *v*] and $D[L,\bot ,\bot ]$ which stores the following. If there is a collection *Q* of alternating cycles and an alternating path with *u* and *v* as endpoints such that $\vert E(Q)\vert =\vert L\vert$ and the edges in *E*(*Q*) are colored with distinct colors from *L*, then we store one such collection in *D*[*L*, *u*, *v*]. Otherwise, we store $\bot$ in *D*[*L*, *u*, *v*]. If there is a collection $Q'$ of alternating cycles such that $\vert E(Q')\vert =\vert L\vert$ and the edges in $E(Q')$ are colored with distinct colors from *L*, then we store one such collection in $D[L,\bot ,\bot ]$. Otherwise, we store $\bot$ in $D[L,\bot ,\bot ]$. We compute the DP table entries in the increasing order of the size of *L*. The base case is when $L=\emptyset$. That is, $D[\emptyset ,\bot ,\bot ]=\emptyset$ and $D[\emptyset ,u,v]=\bot$ for any two vertices *u* and *v*. Now, for any $\emptyset \ne L\subseteq C_i$, and two distinct vertices $u,v\in V(G)$, we compute *D*[*L*, *u*, *v*] and $D[L,\bot ,\bot ]$ as follows. If there is an edge $(x,y)\in E(G)$ such that the color *c* of (*x*, *y*) belongs to *L*, and $D[L\setminus \{c\},x,y]=Q_1\ne \bot$, then we store the graph induced on $E(Q_1)\cup \{(x,y)\}$ in $D[L,\bot ,\bot ]$. Otherwise we store $\bot$ in $D[L,\bot ,\bot ]$. Also, if there a vertex *w* adjacent to *v* such that the color *c* of (*w*, *v*) belongs to *L*, and $D[L\setminus \{c\},u,w]=Q_1'\ne \bot$, then we store the graph induced on $E(Q'_1)\cup \{(w,v)\}$ in *D*[*L*, *u*, *v*]. If $(u,v)\in E(G)$, the color *c* of (*u*, *v*) belongs to *L*, and $D[L{\setminus } \{c\},u,v]=Q'_1\ne \bot$, then we store the graph induced on $E(Q'_1)\cup \{(u,v)\}$ in *D*[*L*, *u*, *v*]. Otherwise we store $\bot$ in *D*[*L*, *u*, *v*]. At the end we output $D[C_i,\bot ,\bot ]$.

We now prove that the computation of $D[C_i,\bot ,\bot ]$ is correct. For this we prove the following statements using induction on |*L*|.

For any $L\subseteq C_i$ and distinct vertices *u* and *v* the following statements hold.

(i) If $D[L,u,v]=Q\ne \bot$, then *Q* is a collection of alternating cycles and an alternating path with *u* and *v* as endpoints such that $\vert E(Q)\vert =\vert L\vert$ and the edges in *E*(*Q*) are colored with distinct colors from *L*.
(ii) If $D[L,u,v]= \bot$, then there is no collection *Q* of alternating cycles and an alternating path with *u* and *v* as endpoints such that $\vert E(Q)\vert =\vert L\vert$ and the edges in *E*(*Q*) are colored with distinct colors from *L*.
(iii) If $D[L,\bot ,\bot ]=Q'\ne \bot$, then $Q'$ is a collection of alternating cycles such that $\vert E(Q')\vert =\vert L\vert$ and the edges in $E(Q')$ are colored with distinct colors from *L*.
(iv) If $D[L,\bot ,\bot ]= \bot$, then there is no collection $Q'$ of alternating cycles such that $\vert E(Q')\vert =\vert L\vert$ and the edges in $E(Q')$ are colored with distinct colors from *L*.

The base case is when $L=\emptyset$ and the we know that $D[\emptyset ,\bot ,\bot ]=\emptyset$ and $D[\emptyset ,u,v]=\bot$. Clearly, the statements above are true for $L=\emptyset$. Now consider the induction step.

*Case 1: *$D[L,u,v]=Q$. From the steps of the algorithm we know that one of the following two statements is true:

- There is an edge $(w,v)\in E(G)$ such that the color *c* of (*w*, *v*) belongs to *L*, and $D[L\setminus \{c\},u,w]=Q'_1\ne \bot$ and $E(Q)=E(Q'_1)\cup \{(w,v)\}$.
- $(u,v)\in E(G)$ such that the color *c* of (*u*, *v*) belongs to *L*, and $D[L{\setminus } \{c\},\bot ,\bot ]=Q_1\ne \bot$ and $E(Q)=E(Q'_1)\cup \{(u,v)\}$.

If statement (a) is true, then by the induction hypothesis, we know that $Q'_1$ is a collection of alternating cycles and an alternating path with *u* and *w* as endpoints such that $\vert E(Q'_1)\vert =\vert L\vert -1$ and the edges in $E(Q'_1)$ are colored with distinct colors from $L\setminus \{c\}$. Then, we get that *Q* is a collection of alternating cycles and an alternating path with *u* and *v* as endpoints such that $\vert E(Q)\vert =\vert L\vert$ and the edges in *E*(*Q*) are colored with distinct colors from *L*. The argument when statement (*b*) is true, is identical to the above case.

*Case 2:* $D[L,u,v]= \bot$. For the sake of contradiction, suppose there is a collection *Q* of alternating cycles and an alternating path with *u* and *v* as endpoints such that $\vert E(Q)\vert =\vert L\vert$ and the edges in *E*(*Q*) are colored with distinct colors from *L*. Let (*w*, *v*) be the edge in *Q* which is incident to *v* and let *c* be the color of (*w*, *v*). Here we consider the case when $u\ne w$, as the other case is symmetric. Note that $E(Q)\setminus \{(w,v)\}$ induces a graph $Q'$ which is a collection of alternating cycles and an alternating path with *u* and *w* as endpoints such that $\vert E(Q')\vert =\vert L\vert -1$ and the edges in $E(Q')$ are colored with distinct colors from $L\setminus \{c\}$. This implies that, by induction hypothesis, $D[L\setminus \{c\},u,w]\ne \bot$. Thus, from the steps of the algorithm, we get that $D[L,u,v]\ne \bot$ which is a contradiction to our assumption.

*Case 3:* $D[L,\bot ,\bot ]=Q'\ne \bot$. The argument for this case is similar to that of case 1.

*Case 2:* $D[L,\bot ,\bot ]= \bot$. The argument for this case is similar to that of case 2.

As the number of table entries for *D*[., ., .] is upper bounded by $2^{rs+1}n^2$, the running time to compute $Q_i$ is $2^{rs} n^{\mathcal {O}(1)}$. We have already mentioned that the cost of guessing $C_1,\ldots ,C_r$ is $2^{r^2s}$. Therefore, the total running time to compute the required *r* perfect matchings is $2^{\mathcal {O}(r^2s)} n^{\mathcal {O}(1)}$. $\square$

Finally, we put together both the lemmas and prove the main theorem of the section.

**Theorem 6** *There is an algorithm that given an instance of* Diverse Perfect Matchings, *runs in time* $2^{2^{\mathcal {O}(kd)}}n^{\mathcal {O}(1)}$ *and outputs the following: If the input is a* No-instance *then the algorithm outputs* No. *Otherwise the algorithm outputs* Yes *with probability at least* $1-\frac{1}{e}$.

### Proof

Let (*G*, *k*, *d*) be the input instance. Our algorithm $\mathcal{A}$ has two steps. In the first step of $\mathcal{A}$ we compute a collection of matchings greedily such that they are far apart using Lemma 6. Towards that first we run an algorithm to compute a maximum matching in *G* and let $M_1$ be the output. If $M_1$ is not a perfect matching we output No and stop. Next we iteratively apply Lemma 6 to compute a collection of perfect matchings that are far apart. Formally, at the beginning of step *i*, where $1\le i<k$, we have perfect matchings $M_1,\ldots ,M_i$ such that $\vert M_{j}\setminus M_{j'}\vert \ge 2^{k-i}d$ for any two distinct $j,j'\in \{1,\ldots ,i\}$. Now, we apply Lemma 6 with $r=i$ and $s=2^{k-i-1}d$ and it will either output a matching $M_{i+1}$ such that $\vert M_{i+1}{\setminus } M_j\vert \ge 2^{k-i-1}d$ for all $j\in \{1,\ldots ,i\}$, or not. If no such matching exists, then the first step of the algorithm $\mathcal{A}$ is complete. So at the end of the first step of the algorithm $\mathcal{A}$, we have perfect matchings $M_1,\ldots ,M_q$, where $q\in \{1,\ldots ,k\}$ such that

(i) for any two distinct integers $i,j\in \{1,\ldots ,q\}$, $|M_i\setminus M_j|\ge 2^{k-q}d$, and
(ii) if $q\ne k$, then for any other perfect matching $M\notin \{M_1,\ldots ,M_q\}$, $\vert M{\setminus } M_j\vert \le 2^{k-q-1}d$.

If $q= k$, then $\{M_1,\ldots ,M_k\}$ is a solution to the instance (*G*, *k*, *d*), and hence our algorithm $\mathcal{A}$ outputs Yes. Now on, we assume that $q\in \{1,\ldots ,k-1\}$. Statements (*i*) and (*ii*), and Observation 4 imply that

(i) for any two distinct integers $i,j\in \{1,\ldots ,q\}$, $|M_i\bigtriangleup M_j|\ge 2^{k-q+1}d$, and
(iv) for any perfect matching $M\notin \{M_1,\ldots ,M_q\}$, $\vert M\bigtriangleup M_j\vert < 2^{k-q}d$.

Statements (*ii*) and (*iv*), and Observation 3 imply the following claim.

### Proposition 14

For any perfect matching *M*, there exists a unique $i\in \{1,\ldots ,q\}$ such that $\vert M\bigtriangleup M_i\vert < 2^{k-q}d$.

Let $\mathcal{M}=\{M_1^{\star },\ldots ,M^{\star }_k\}$ is a solution to the instance (*G*, *k*, *d*). Then, by Proposition 14, there is a partition of $\mathcal{M}$ into $\mathcal{M}_1\uplus \ldots \uplus \mathcal{M}_q$ (with some blocks possibly being empty) such that for each $i\in \{1,\ldots ,q\}$, and each $M\in \mathcal{M}_i$, $\vert M\bigtriangleup M_i\vert \le 2^{k-q}d$. Thus, in the second step of our algorithm $\mathcal{A}$, we guess $r_1=\vert \mathcal{M}_1\vert ,\ldots , r_q=\vert \mathcal{M}_q\vert$ and apply Lemma 7. That is, for each $i\in \{1,\ldots ,q\}$ such that $r_i\ne 0$, we apply Lemma 7 with $M=M_i$, $r=r_i$, and $s=2^{k-q}d$. Then for each $i\in {1,\ldots ,q}$, let the output of Lemma 7 be $N_{i,1},\ldots ,N_{r_i}$. Clearly $\vert N_{i,j}\bigtriangleup N_{i,j'}\vert \ge d$ for any two distinct $j,j'\in \{1,\ldots ,r_i\}$. Observation 3 and statement (*iii*) implies that for any two distinct $i,j\in \{1,\ldots ,q\}$, the cardinality of the symmetric difference between a matching in $\{N_{i,1},\ldots ,N_{i,r_i}\}$ and a matching in $\{N_{j,1,\ldots ,N_{j,r_j}}\}$ is at least *d*.

If algorithm $\mathcal{A}$ computes a solution in any of the guesses for $r_1,\ldots ,r_d$, then we output Yes. Otherwise we output No. As the number of choices for $r_1,\ldots r_k$ is upper bounded by $k^{\mathcal {O}(k)}$, from Lemma 6 and Lemma 7 we get that the running time of $\mathcal{A}$ is $2^{2^{\mathcal {O}(kd)}}n^{\mathcal {O}(1)}$ and the success probability is at least $2^{-2^{ckd}}$ for some constant *c*. To get success probability $1-1/e$, we do $2^{2^{ckd}}$ many executions of $\mathcal{A}$ and output Yes if we succeed in at least one of the iterations and output No otherwise. Thus, the running time of the overall algorithm is $2^{2^{\mathcal {O}(kd)}}n^{\mathcal {O}(1)}$.$\square$

## Conclusion

In this work we take up weighted diverse variants of two classical matroid problems and the unweighted diverse variant of a classical graph problem. We first show that the two diverse matroid problems are ${{\,\mathrm{\textsf{NP}}\,}}$-hard, and that the diverse graph problem cannot be solved in polynomial time even for the smallest sensible measure of diversity. We then show that all three problems are ${{\,\mathrm{\textsf{FPT}}\,}}$ with the combined parameter (*k*, *d*) where *k* is the number of solutions and *d* is the diversity measure.

We conclude with a list of open questions:

- In this work we show that the unweighted, counting variant of Weighted Diverse Bases does not have a polynomial-time algorithm unless ${{\,\mathrm{\textsf{P}}\,}} ={{\,\mathrm{\textsf{NP}}\,}}$ (Theorem 7). This is the case when all the weights are 1 and $d=1$ or $d=2$. Both the weighted and unweighted variants can be solved in polynomial time when $k=1$ (the greedy algorithm) and $k=2$ ((weighted) matroid intersection). What happens for larger, constant values of *d* and/or *k*? Till what values of *d*, *k* does the problem remain solvable in polynomial time? These questions are interesting also for special types of matroids. For instance, is there a polynomial-time algorithm that checks if an input graph has *three* spanning trees whose edge sets have pairwise symmetric difference at least *d*, or is this already ${{\,\mathrm{\textsf{NP}}\,}}$-hard?
- A potentially easier question along the same vein would be: we know from Theorem 7 that Weighted Diverse Bases is unlikely to have an ${{\,\mathrm{\textsf{FPT}}\,}}$ algorithm parameterized by *d* alone. Is Weighted Diverse Bases ${{\,\mathrm{\textsf{FPT}}\,}}$ parameterized by *k* alone?
- By the results of Bérczi, Csáji, and Király [4], we have that Weighted Diverse Common Independent Sets is already ${{\,\mathrm{\textsf{NP}}\,}}$-hard for unit weights and $k=2$. Thus, the problem is para-NP-hard when parameterized by *k*. Is Weighted Diverse Common Independent Sets ${{\,\mathrm{\textsf{FPT}}\,}}$ when parameterized by *d*?

## Footnotes

1 See Theorem 7 for an alternative hardness result for Weighted Diverse Bases.

2 Compare with Theorem 1.
